# Use of near-infrared spectroscopy for screening the oil content, protein, phytic acid, glucosinolates, and fatty acid profile in oilseed *Brassica* species

**DOI:** 10.3389/fnut.2025.1632421

**Published:** 2025-09-02

**Authors:** Anubhuti Sharma, Purnima Sogarwal, Arun Kumar, Ramesh

**Affiliations:** ^1^Biochemistry Laboratory, Indian Council of Agriculture Research-Indian Institute of Rapeseed Mustard Research (ICAR-IIRMR), Bharatpur, Rajasthan, India; ^2^Biotechnology Division, GLA University, Mathura, Uttar Pradesh, India; ^3^Agricultural Research Station, Mandor, Agriculture University, Jodhpur, Rajasthan, India

**Keywords:** spectroscopy, seed quality traits, *Brassica* accessions, calibration models, prediction efficiency, Pearson correlation, FT-NIR

## Abstract

The escalating global demand for vegetable oils underscores the need to enhance the quality and yield of oilseed crops with *Brassica* species, due to their rich oil content and nutritional benefits. Traditional methods for assessing seed quality traits are often slow and destructive, limiting their scalability in breeding programs. This study presents Fourier transform near-infrared (FT-NIR) spectroscopy as a rapid, non-destructive alternative to evaluate these critical traits across 80 diverse *Brassica* genotypes, including three species, namely, *Brassica juncea, Brassica napus*, and *Brassica rapa*. By integrating FT-NIR with principal component analysis and partial least squares regression, we developed robust calibration models, achieving high predictive accuracy (R^2^ > 0.85 for key fatty acids; R^2^ = 0.92 for oil content) and low error rates (MAE < 1.8). Our results revealed significant genetic variability, with oil content showing remarkable stability (CV = 0.68%) and erucic acid exhibiting the highest variation (CV = 9.18%), offering promising avenues for targeted breeding. PCA elucidated 68% of the total variance, spotlighting oleic acid, erucic acid, and oil content as key drivers of genetic differentiation. Pearson correlation analysis also revealed a strong inverse relationship between oleic acid and erucic acid, suggesting potential genetic linkages that could be exploited in breeding programs. The FT-NIR models demonstrated superior throughput and reliability compared to conventional wet chemistry. These findings not only streamline seed quality assessment but also pave the way for breeding *Brassica* cultivars with optimized nutritional profiles high in beneficial polyunsaturated fatty acids and low in anti-nutritional factors.

## 1 Introduction

Rapeseed mustard (*Brassica* spp.) is one of the major oilseed crops in India owing to its high oil content and nutritive values (1). Its oil is used as a favorite cooking oil, mainly in the eastern and northwestern parts of India. The major fatty acids of rapeseed mustard oil are palmitic acid, stearic acid, oleic acid, linoleic acid, linolenic acid, eicosenoic acid, and erucic acid, which determine its nutritional properties. However, a high amount of erucic acid was reported to cause myocardial lipidosis (2). Mustard meal, which is a byproduct of oil extraction, is a valuable source of quality proteins for human consumption and animal fodder. The nutritive value of seed meal is limited by the presence of a few antinutritional compounds, such as glucosinolates and phytic acid. Glucosinolates are antinutritional compounds as they reduce feed palatability which limits the use of defatted meal as a source of nutrients in animal feed (1). Phytic acid reduces the bioavailability of minerals, especially iron, zinc, and calcium, by binding to them in the digestive tract (3).

Rapeseed-mustard varietal improvement program includes the development of varieties with low erucic acid level up to 2% and glucosinolate content up to 30 μ moles/g defatted seed meal. These *Brassica* improvement programs require the screening of a large number of samples from various segregating generations and help in breeding programs to enhance the oil quality of *Brassica* species (4). Therefore, a rapid and non-destructive technique is required for the detection of nutritional and antinutritional parameters in rapeseed mustard quality breeding programs.

Over the past few years, classical analytical methods were used to detect the quality parameters of rapeseed mustard, such as the Soxhlet extraction for oil content, gas chromatography (GC) for fatty acids, Kjeldahl method for crude protein content, tetrachloropalladate method for glucosinolates, and spectrophotometric analysis of phytic acid (5, 6). However, these methods are generally time consuming, cumbersome, destructive, and require skilled operation.

Recent technological advances have brought a rapid, non-destructive, and highly sensitive analytical technique termed near-infrared reflectance (NIR) spectroscopy. NIR operates in the near-infrared region of the spectrum, and it is associated with overtones and combination bands of molecular vibrations, as well as electronic transitions (7). Over recent years, Fourier transform near-infrared (FT-NIR) spectroscopy has emerged as a robust technique for the rapid and non-invasive measurement of seed quality characteristics (8). FTIR operates in the mid-infrared (MIR) region, which is associated with the fundamental vibrational and rotational modes of chemical bonds within molecules. The combination of FT-NIR spectroscopy and chemometrics (multivariate data analysis techniques for biochemical traits) could be widely used in the cereal, oilseed, dairy, horticulture, and other processing industries to predict the chemical composition of biological products with high accuracy (9). Fourier transform infrared spectroscopy (FT NIR) provides quantitative and qualitative analytical information using the multivariate statistical methods (PCA, HCA, LDA, and SIMCA) and multivariate calibration (PCAR and PLS-R) methods to extract information from analytical data and to study the diversity of *Brassica* species (7, 10). FTIR is generally considered more sensitive than NIR spectroscopy for certain types of chemical bonds and functional groups.

To date, no attempt has been made in India to simultaneously determine oil content, total protein, phytic acid, total glucosinolates, and fatty acid profile (palmitic acid, stearic acid, oleic acid, linoleic acid, linolenic acid, eicosenoic acid, and erucic acid) based on intact seeds by FT-NIRS in rapeseed mustard. The aim of this study was to create an FT-NIR calibration model for the rapid, non-destructive assessment of key components in *Brassica* species (mainly diverse genetic germplasms of *Brassica juncea, Brassica napus*, and *Brassica rapa*), including fatty acids, oil content, glucosinolates, phytic acid, and protein.

This study will support breeders in selecting high polyunsaturated fatty acids (PUFA), monounsaturated fatty acids (MUFA), low erucic acid, and low glucosinolate genotypes under varietal developmental programs.

This study will supplement previous research that validated the applicability of FT-NIR spectroscopy on various seed quality features in crop species. The findings of the present study have significant implications for future *Brassica* breeding strategies, as this study will provide a new and more effective research method based on multiple seed quality parameters.

By integrating Fourier transform near-infrared (FT-NIR) spectroscopy with principal component analysis (PCA) and regression analysis, the extent of genetic differentiation among genotypes was evaluated. Fourier transform infrared spectroscopy (FT-NIR) provides quantitative and qualitative analytical information using the multivariate statistical methods (PCA, HCA, LDA, and SIMCA) and multivariate calibration (PCAR and PLS-R) methods to extract information from analytical data and to study the diversity of *Brassica* species (7).

## 2 Materials and methods

Primarily, 100 *Brassica* genotypes were used for NIR spectra readings, and then, they were subjected to the chemometric analysis of different biochemical parameters in the laboratory. Out of these 100 samples, 80 were used for final model calibration in FT-NIR because 20 samples were found unsuitable from the chemometric analysis due to high moisture and physical impurities. A total of 80 *Brassica* seed samples consisting of 40 accessions, 62 advanced breeding lines, and 18 varieties were evaluated for their seed quality traits, including oil profile, fatty acid profile, protein, glucosinolate content, and phytic acid content. The accessions of *B. juncea, B. napus*, and *B. rapa* were obtained from the germplasm division of ICAR-IIRMR, Bharatpur, India.

The specific areas of the states from which the seed samples were taken were systematically identified based on the cultivation history and the possibility of capturing variability was considered. The advanced breeding lines used in this study were those genotypes that were tested under the All India Coordinated Research Project (AICRP) program and grown in a randomized block design (RCBD) with three replications during the rabi harvest, 2021–2023. Samples were taken from released varieties maintained by the germplasm division of ICAR-IIRMR, Bharatpur, India.

### 2.1 Compositional analysis by reference methods

The chemometric analysis was carried out in the laboratory for oil, protein, phytic acid, glucosinolate, erucic acid, linoleic acid, oleic acid, linolenic acid, palmitic acid, stearic acid, and eicosenoic acid. The oil content (%) was measured using the Soxhlet extraction method (6). The protein content (%) was estimated by determining the nitrogen content using Kjeldahl method and multiplying with the factor 6.25 (11). Phytic acid estimation was carried out according to the instructions of Verma et al. (6). Glucosinolate estimation was based on a spectrophotometric method (12). All fatty acid content was determined through gas–liquid chromatography (Nucon Model 5765, New Delhi, India) equipped with SP 2300 + 2310 SS column, following the procedure of fatty acid methyl esters development by Vasudev et al. (13).

In total, three *Brassica* species (*B. juncea, B. napus*, and *B. rapa*) including check varieties, were collected. For spectra data acquisition and analysis, seed samples in bulk were placed in a sample cup of 5 cm diameter at the beam outlet of the FT-NIR spectrometer (Thermo Fisher Scientific, Model no. Antaris II DR, Serial no.AHY2211830, USA). For each sample, the spectrum was recorded in total reflectance mode in the wavelength ranging from 4,000 to 12,000 cm^−1^ by averaging 64 scans with ~1 min of analysis.

For the estimation of oil content, raw spectra were processed through the standard normal variate transformation (SNV) method, whereas all other remaining parameters were estimated after the processing of raw spectra through the PLS method. Second derivatives within the limits were used to estimate oil content, palmitic acid, stearic acid, oleic acid, linoleic acid, linolenic acid, eicosanoid acid, and erucic acid. Protein content, phytic acid, and glucosinolate content were estimated through the first derivatives within the selected spectral range. Following the assessment made on preprocessing techniques, SNV preceded by a first derivative with a Norris derivative filter (5-point window, interval of 3) was selected for further analysis since it was observed to present the highest balance between noise reduction and the generation of spectrum features.

### 2.2 Development and evaluation of the NIR calibration models

Spectra were first treated with detrending and standard normal variate transformation to minimize scattering effects and reduce particle size noise. Partial least squares regression, as a linear chemometric algorithm, was used to build calibration and cross-validation models.

The outliers were identified as chemical outliers when a point was outside ±3SDs of calibration y-residuals, which indicated that these samples could not be used to achieve the best, most reliable model possible. Therefore, 20 samples out of 100 were rejected. Consequently, the remaining samples (80) were used for calibration, as indicated in Table 1.

**Table 1:** Descriptive statistics of seed quality traits in *Brassica* genotypes (A, B, C) (Actual: laboratory analyzed; Cal, calculated; Val, validated).

**Biochemical**	**Oil (%)**			**Protein (%)**			**Glucosinolate** μ**mole/g**			**Phytic acid (%)**		
**Descriptive statistics**	**Actual**	**Cal**	**Val**	**Actual**	**Cal**	**Val**	**Actual**	**Cal**	**Val**	**Actual**	**Cal**	**Val**
Mean	39.1	38.66	38.54	27.2	26.55	26.42	50.6	49.85	49.84	2.7	2.96	2.94
Minimum	34.9	34.53	33.50	11.0	10.37	10.35	10.9	10.36	10.32	1.5	1.54	1.55
Maximum	41.5	40.75	40.72	37.4	36.75	35.74	116.4	115.75	115.72	3.9	3.61	3.60
SD	1.5	0.25	0.21	8.4	0.24	0.23	31.1	30.59	30.58	0.8	0.30	0.29
CV	2.1	-	-	4.6	-	-	3.5	-	-	3.3	-	-
No. of genotypes	80	62	9	80	60	7	80	65	8	80	64	8
MAE		0.35	0.34		0.42	0.41	-	3.76	3.74	-	0.28	0.27
R^2^		0.920	0.934		0.966	0.943	-	0.986	0.973	-	0.749	0.8911
RPD		2.85	2.49		2.67	2.84	-	2.38	2.49	-	2.26	2.25
RMSEC		0.486			1.80		-	4.61	-	-	0.43	-
RMSEP			0.540			2.78	-	-	3.91	-	-	0.59
**Fatty acid**	**Palmitic acid**			**Stearic acid (%)**			**Oleic acid (%)**			**Linoleic acid (%)**		
Mean	4.0	3.89	3.95	2.1	1.66	1.67	36.9	36.46	36.45	31.8	31.37	32.38
Minimum	2.0	1.86	1.78	0.8	0.29	0.28	9.7	9.26	8.75	14.3	13.67	13.66
Maximum	5.4	4.87	4.86	3.1	2.91	2.20	51.9	50.97	50.96	43.2	42.77	42.76
SD	0.9	0.8	0.8	0.6	0.36	0.35	12.3	12.45	11.20	7.7	7.28	7.29
CV	8.2	-	-	17.0	-	-	7.5	-	-	4.5	-	-
No. of genotypes	80	54	13	80	50	8	80	55	8	80	53	9
MAE	-	0.14	0.13	-	0.16	0.17	-	1.15	1.14	-	1.8	1.5
R^2^	-	0.996	0.931	-	0.832	0.885	-	0.895	0.858	-	0.867	0.919
RPD	-	2.51	2.49	-	2.40	2.41	-	2.43	2.42	-	2.47	2.46
RMSEC	-	0.06	-	-	0.15	-	-	4.82		-	4.01	-
RMSEP	-	-	0.38	-	-	0.17	-		4.80	-	-	1.90
	**Linolenic acid**			**Eicosenoic acid (%)**			**Erucic acid (%)**					
Mean	15.2	12.11	15.38	2.8	1.96	1.97	9.6	8.83	8.32			
Minimum	9.1	7.27	8.36	0.2	0.1	0.1	0.2	0.1	0.1			
Maximum	36.2	36.92	32.53	10.7	10.43	10.42	49.7	48.97	48.96			
SD	9.1	8.58	9.38	3.6	2.58	2.87	16.7	15.25	15.24			
CV	10.7	-	-	22.4	-	-	22.4	-	-			
No. of genotypes	80	59	8	80	57	10	80	60	8			
MAE	-	3.3	3.9	-	0.24	0.23	-	1.57	1.56			
R^2^	-	0.911	0.898	-	0.834	0.827	-	0.898	0.773			
RPD	-	2.36	2.35	-	2.29	2.28	-	2.12	2.11			
RMSEC	-	4.21	-	-	0.13	-	-	3.61	-			
RMSEP	-	-	5.03	-	-	0.12	-	-	4.43			

CV, coefficient of variation; SD, standard deviation; R^2^, coefficient of determination; MAE, mean absolute error; RPD, ratio of performance to deviation; RMSEC, root mean square error of calibration; RMSEP, root mean square error of prediction.

Applied mathematical transformations, such as derivatives, multiple scatter corrections, smoothening, were used for data pre-processing to enhance spectral features and eliminate or minimize undesired variations in the NIR spectra. The obtained dataset of each trait was then correlated with laboratory-analyzed data using partial least squares regression. The calibration process was carried out using the TQ Analyst software (v 9.13.292, Thermo Fisher Scientific Inc., USA). The performance of the developed model was assessed by an external validation procedure to determine the accuracy and precision of the equations derived during calibration for each trait in all samples. To assess the precision of the equations, various statistical measures were employed.

The calibration of the obtained spectral data was carried out according to partial least squares calibration or standard normal variate transformation for each of the seed quality attributes to be predicted. With regard to these models, some data were employed for the training or calibration of the models, while others were used for the testing or validation of the models. When comparing the accuracy of the models in prediction, there are certain measures that were employed, and they are the coefficient of determination R^2^, the mean absolute error (MAE), and the ratio of performance to deviation (RPD) (14).

Several statistical terms were used to evaluate the performance of NIRS models, including the Coefficient of Determination for Calibration (R^2^C), Coefficient of Determination for Cross-Validation (${\text{R}}_{\text{CV}}^{2}$), Standard Error of Calibration (SEC), Standard Error of Cross-Validation (SECV), and Residual Predictive Deviation (RPD) (15).

The statistical formulas are as follows:


(1) $$\begin{array}{c} {\text{r}}^{2}=\frac{\sum_{\text{i}=1}^{\text{n}}\left(\text{y}-{\bar{\text{y}}}^{2}\right)}{\sum_{\text{i}=1}^{\text{n}}\left({\text{y}}_{\text{i}}-{\bar{\text{y}}}^{2}\right)} \end{array}$$

(2) $$\begin{array}{c} \text{RMSEP}=\sqrt{\frac{\sum {\left({\text{Y}}_{\text{pred}}-{\text{Y}}_{\text{ref}}\right)}^{2}}{\text{n}}} \end{array}$$

(3) $$\begin{array}{c} \text{Bias}={\bar{\text{Y}}}_{\text{pred}}-{\bar{\text{Y}}}_{\text{ref}} \end{array}$$

(4) $$\text{SEE=}\sqrt{\frac{\text{n}}{\text{n-1}}}\;({\text{RMSEP}}^{\text{2}}{\text{-Bias}}^{\text{2}})$$

(5) $$\begin{array}{c} \text{RPD}=\text{SD}/\text{SEP}, \end{array}$$

where, *y*, NIR measured value; $\bar{y}$, mean “*y*” value for all samples; *yi*, laboratory reference value for the *i*^th^ sample; RMSEP, root mean square error of prediction; *Y*_pred_, predicted value; *Y*_ref_, reference value with standard analysis, *n*, number of samples; Bias, total differences between predicted and reference values. RPD, relative prediction deviation; SD, standard deviation; SEP, standard error of prediction or performance (16).

### 2.3 Statistical analysis

Other statistics that were estimated included the mean, standard deviation, and coefficient of variation (CV) of the characteristics. Eigen analysis was used in this study to determine sources of diversity among the genotypes, while the correlation analysis used a Pearson correlation matrix to determine the extent of the relationship between the various attributes. The coefficient of determination represents explained variance. Standard error indicates the standard deviation of residuals. RPD, calculated as SD/SECV, is a dimensionless statistic that can be used for model evaluation. Therefore, to prove or disprove this hypothesis, the analysis of variance was conducted to analyze the analysis of variance (ANOVA) of each genotype for the different characteristics.

## 3 Result

In this study, 80 *Brassica* genotypes were assessed for seed quality parameters (oil content, fatty acid compositions, protein content, and anticancer factors such as glucosinolates and phytic acid). Table 1 displays the mean, range, standard deviation (SD), and coefficient of variation (CV) for each feature. The ANOVA results highlight the significant differences among the *Brassica* genotypes for each trait (Table 2).

**Table 2:** ANOVA results for seed quality traits.

**Trait**	**PC1**	**PC2**	**GCV (%)**	**PCV (%)**	**GCV/PCV ratio**	***F*-value**	***P*-value**	**Significance**
Palmitic acid (%)	0.321	0.198	20.75	22.32	0.93	4.56	0.001	Significant
Stearic acid (%)	0.174	0.289	20.94	27.01	0.78	3.78	0.002	Significant
Oleic acid (%)	0.635	0.123	32.35	33.21	0.97	9.34	< 0.001	Highly significant
Linoleic acid (%)	0.512	0.341	23.76	24.18	0.98	5.67	0.001	Significant
Linolenic acid (%)	0.298	0.564	22.34	24.79	0.90	3.45	0.004	Significant
Eicosanoic acid (%)	0.267	0.276	21.30	22.40	0.88	2.01	0.001	Significant
Erucic acid (%)	0.614	0.218	123.88	125.89	0.98	15.23	< 0.001	Highly significant
Glucosinolate (μmol/g)	0.342	0.431	61.56	61.66	1.00	8.56	< 0.001	Highly significant
Protein (%)	0.412	0.267	30.55	30.89	0.99	6.12	< 0.001	Highly significant
Oil content (%)	0.652	−0.133	3.08	3.7	0.83	7.78	< 0.001	Highly significant
Phytic acid (mg/g)	0.321	−0.478	9.61	27.29	0.35	4.89	0.002	Significant

These results show that considerable differences exist among the *Brassica* genotypes for these traits, and the degree of variability appears to be high for most characters, especially for oil content, oleic acid, and erucic acid. High CV values of linoleic acid (10.7%), eicosenoic acid, and erucic acid (22.4%) indicate that there may be heterogeneity in replicates and that outliers may be identified and removed.

However, the CV for the oil content and other parameters was very low, with a uniform distribution among replicates. This finding indicates that oil content is a stable characteristic and also illustrates the accuracy of FT-NIR spectroscopy in its quantification.

### 3.1 Descriptive statistics

The mean oil content of 80 *Brassica* samples was 39.1% (laboratory), 38.66% (calibration, 62 genotypes), and 38.54% (validation, 9 genotypes), with a coefficient of variation (CV) of 2.1%, R^2^ of 0.920–0.934, and RPD of 2.49–2.85, indicating strong FT-NIR predictive accuracy (MAE: 0.34–0.35, root mean square error of calibration (RMSEC): 0.486, and root mean square error of prediction (RMSEP): 0.540). Protein content averaged 27.2% (laboratory), 26.55% (calibration, 60 genotypes), and 26.42% (validation, 7 genotypes), with R^2^ of 0.966–0.943 and RPD of 2.67–2.84. Glucosinolate mean was 50.6 μmol/g (laboratory), 49.85% (calibration, 65 genotypes), and 49.84% (validation, 8 genotypes), with R^2^ of 0.986–0.973 and RPD of 2.38–2.49. Phytic acid averaged 2.7% (laboratory), 2.96% (calibration, 64 genotypes), and 2.94% (validation, 8 genotypes), with R^2^ of 0.749–0.891 and RPD of 2.25–2.26. Fatty acids like oleic (36.9%) and linoleic (31.8%) showed R^2^ of 0.858–0.996 and RPD of 2.42–2.51 across calibrations and validations, demonstrating robust model performance for seed quality traits in mustard genotypes (Table 1).

### 3.2 Genetic coefficient of variation (GCV) and phenotypic coefficient of variation (PCV) and Pearson correlation matrix for seed quality traits

The genetic coefficient of variation and the phenotypic coefficient of variation values, ratios, F-values, and *P*-values for the key traits provide insight into the genetic and environmental contributions to trait variability (Table 2).

**Figure 1:**
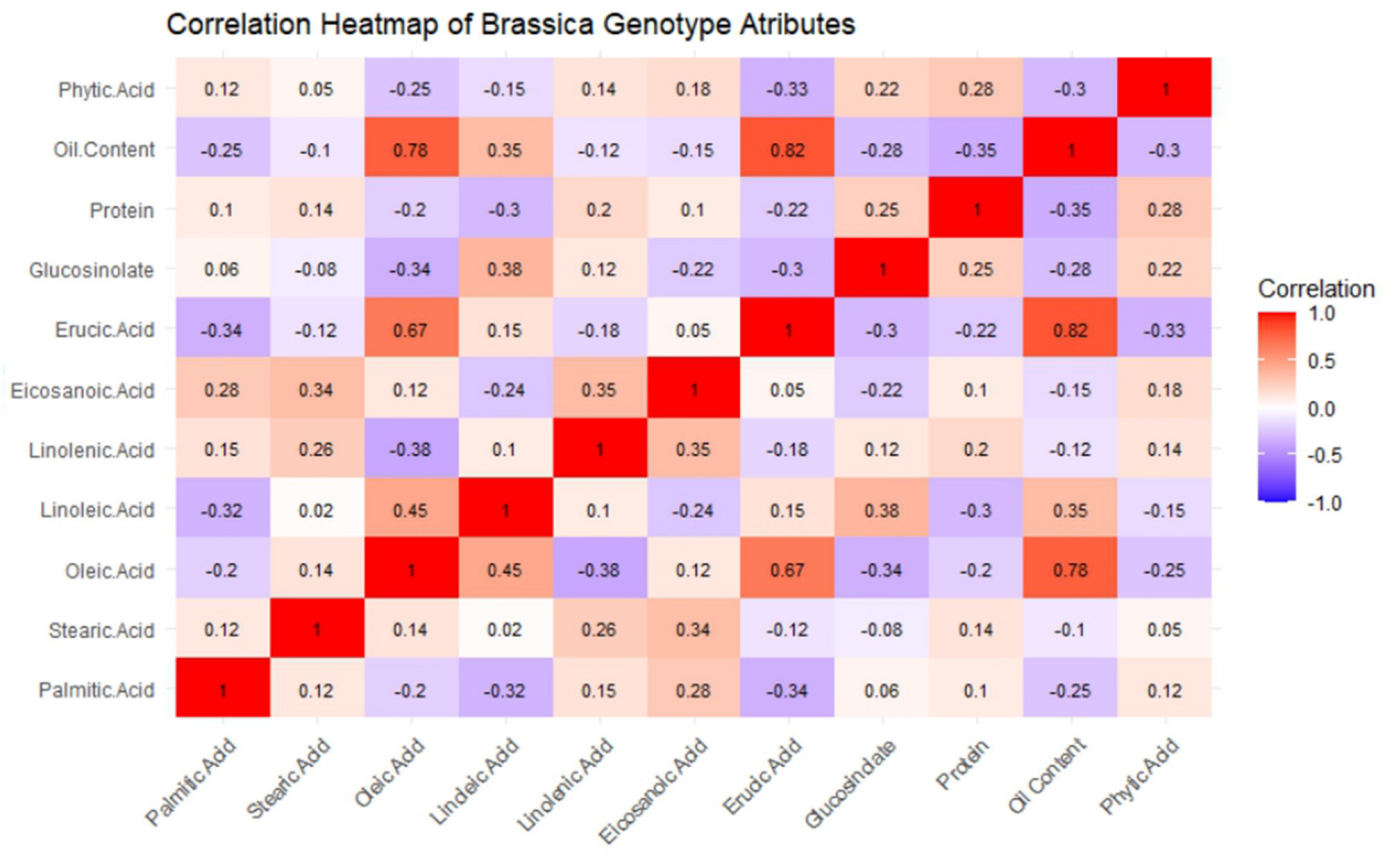
Heat map with Pearson correlation matrix between samples of mustard seeds' biochemical parameters.

The GCV and PCV values indicate that a few traits, such as erucic acid, are genetically controlled because the ratios of GCV and PCV were high. Figure 1 shows the coefficients of the related parameters of seed quality, which enables us to reveal the existing and possible genetic correlations. According to the Pearson correlation matrix mentioned, oleic acid and erucic acid are inversely correlated. Furthermore, such traits as diversity of habit may also be influenced by closely linked genetic factors. Similarly, the table underscores trade-off associations, for example, between the negative correlation of oil content with palmitic acid and phytic acid.

**Figure 2:**
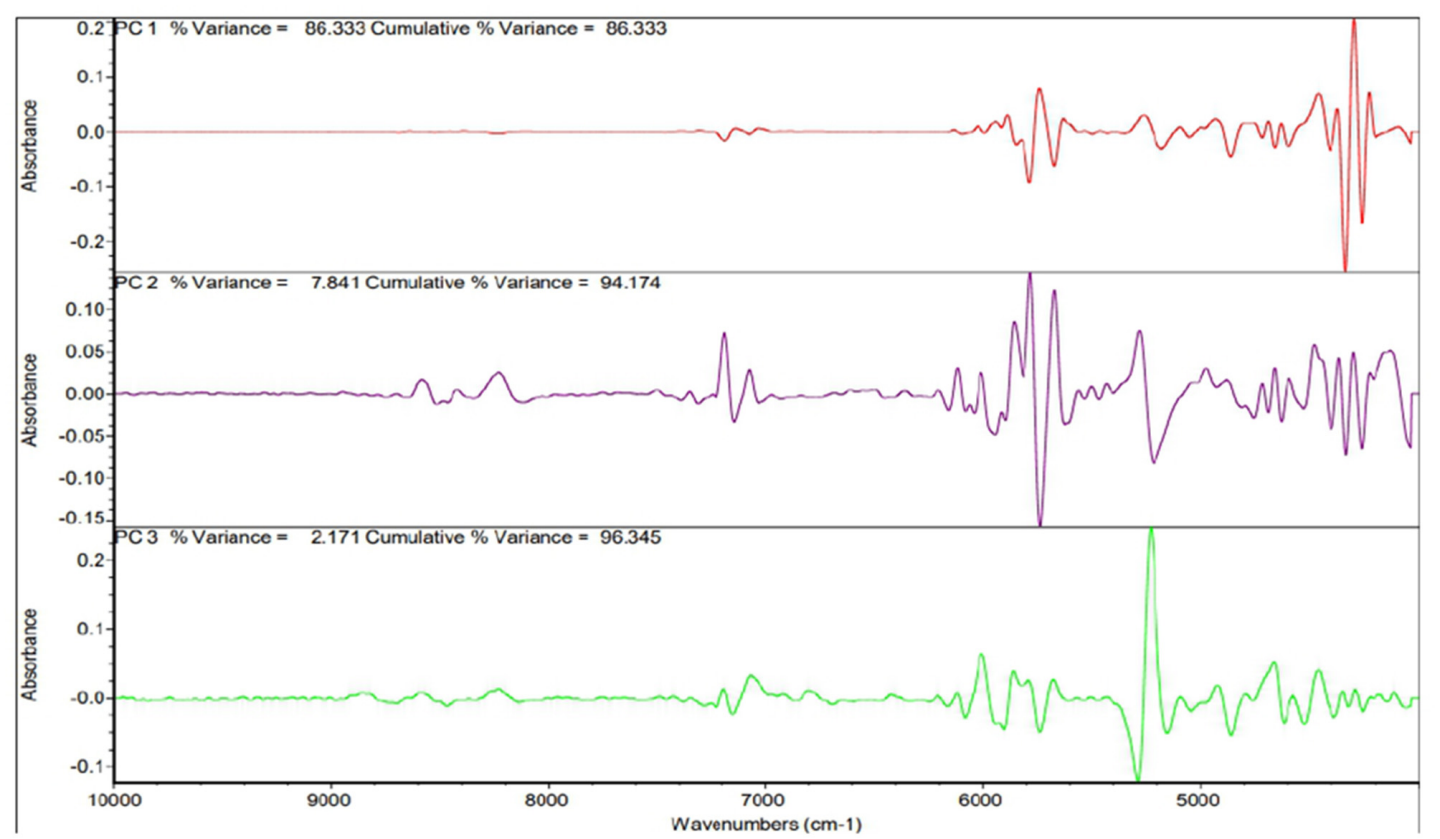
Post-processing PCA spectra present the whole spectrum contribution of the main parts (PC1–PC3).

**Figure 3:**
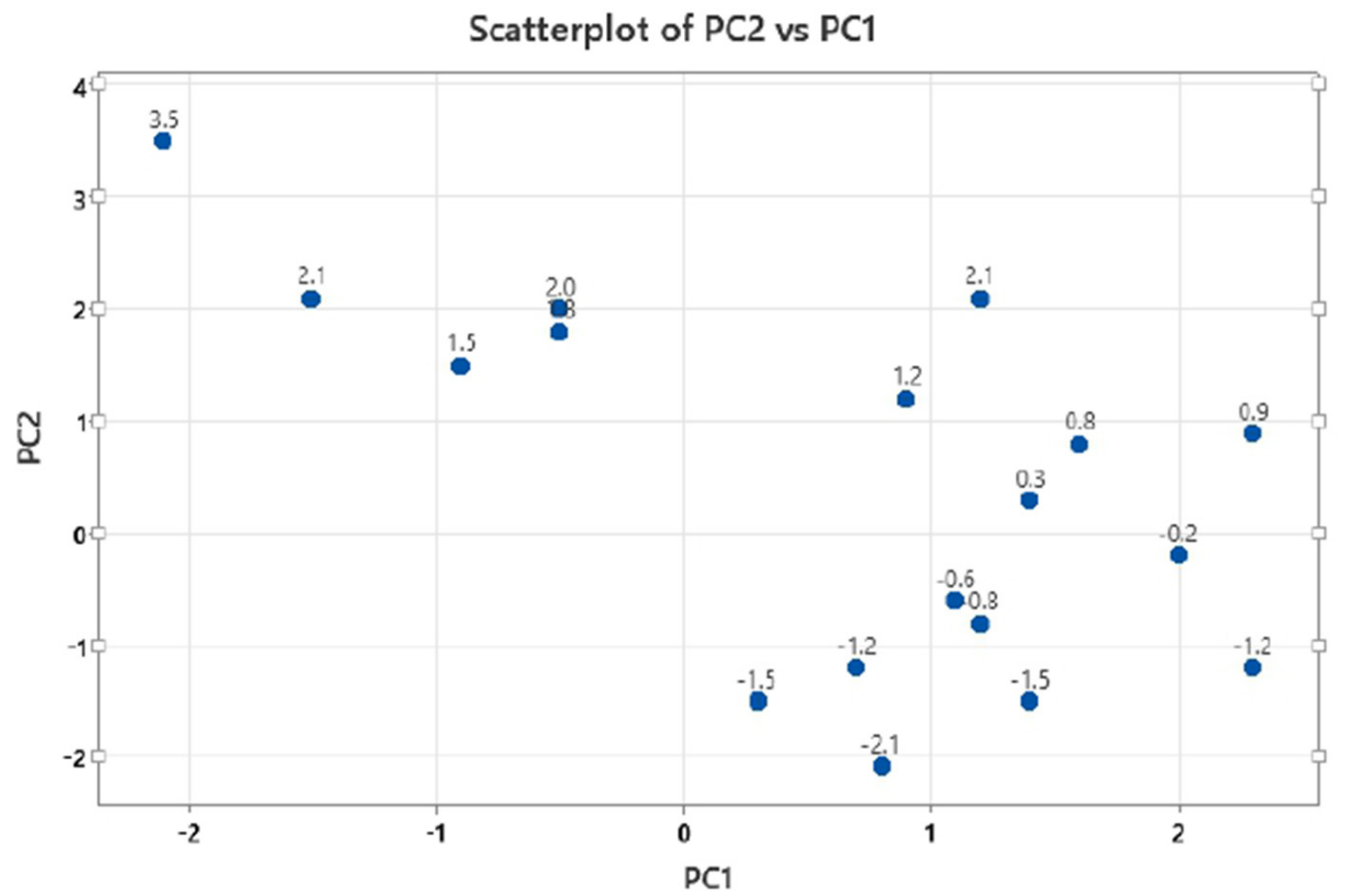
Multivariate distribution of mustard seed samples with principal component 2 (PC2) vs. principal component 1 (PC1) scatter plot.

**Figure 4:**
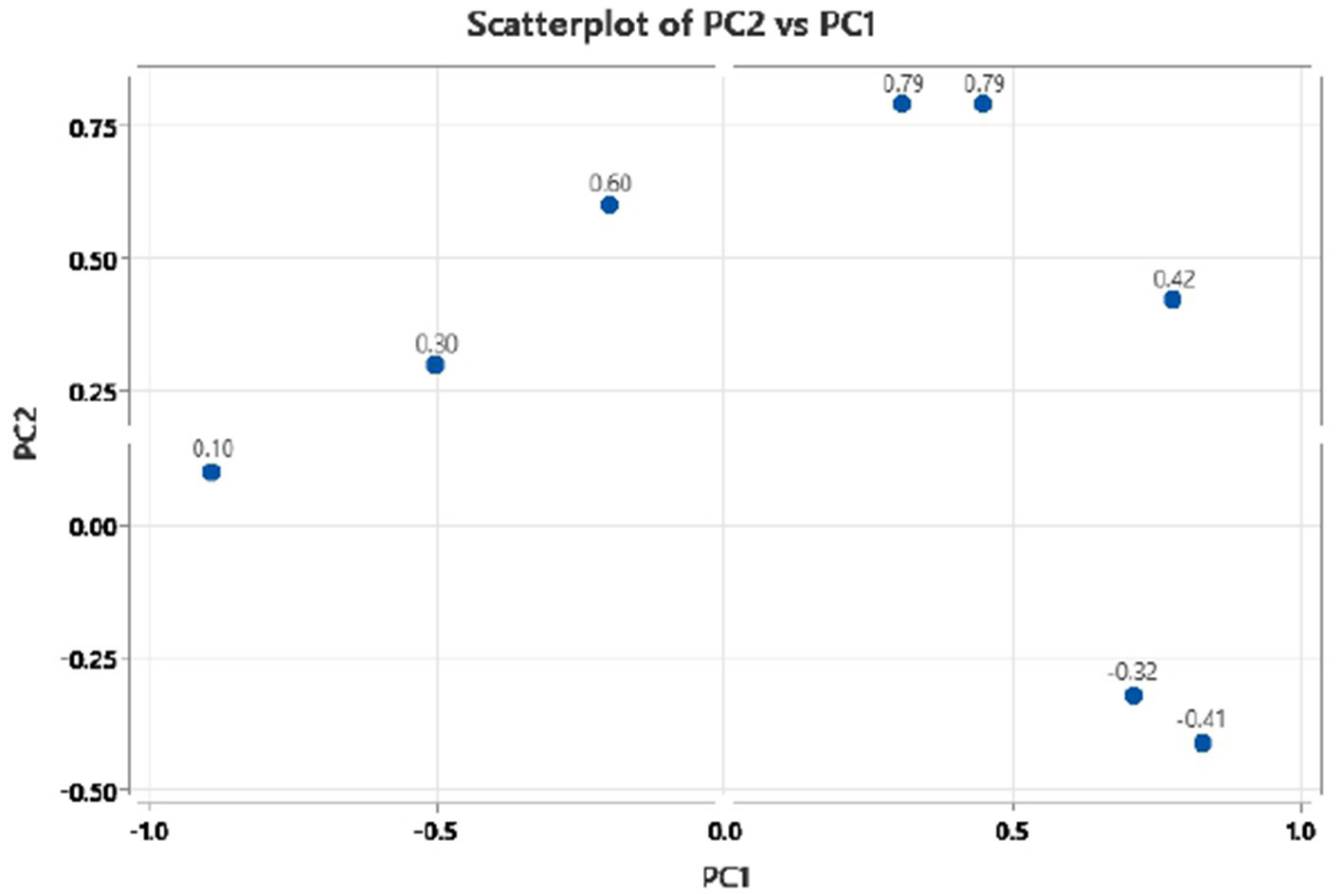
Principal component 2 (PC2) vs. principal component 1 (PC1) scatter plot of mustard seed components in PCA biplot presenting variable loadings.

**Figure 5:**
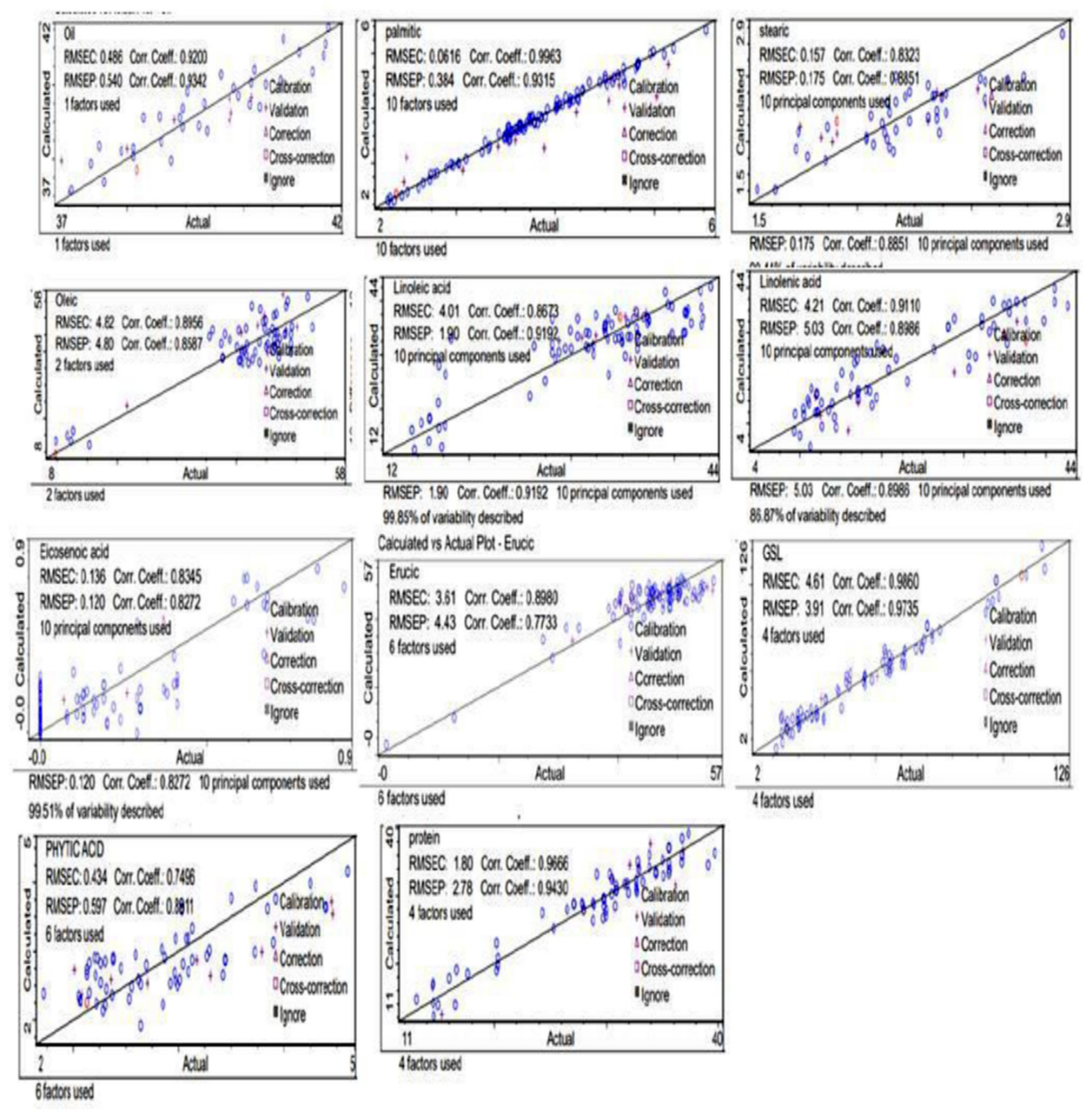
Protein, glucosinolate, fatty acids, oil, and phytic acid predicted against reference plots.

The principal component analysis (Figure 2) was carried out to show the relation between the different seed quality indicators and to determine if there exists any group or pattern among the genotypes. PC1 represents the spectral data variation in the range of 5,000–4,000 cm^−1^, indicating significant contributions from overtones and combination bands, which are typically associated with O–H, N–H, and C–H bonds in oils and proteins. PC2 captures secondary variations, possibly due to differences in fatty acid composition or minor chemical constituents. PC3 exhibits minor variance and represents small sample differences (Figures 3, 4). A distinct sharp peak near 5,000 cm^−1^, possibly related to a minor functional group or instrumental artifact. Together, the first two principal components (PC1 and PC2) explained 68% of total variability. PC1 and PC2 both explain the diversions of all the traits. It is clear from Figures 3, 4 that all fatty acids are present in a similar range of the spectra. Multiple regression plots from a partial least squares (PLS) regression model (Figure 5) indicate the relationship between actual vs. predicted values of specific nutritional components in *Brassica* seed samples.

### 3.3 Loading score of principal components for seed quality traits

Table 2 provides information on PCA loading scores, which depict the extent to which each character contributes to the two main components. The findings from the PCA loading values show that among the parameters that are indicators of genetic variation in the genotypes are oleic acid, erucic acid, and oil content. Out of these traits, oleic acid has a high positive loading on the first component, confirming that they account for the majority of the variance in the data.

## 4 Discussion

The assessed attributes included oil content and fatty acid profiles (C 16:0, C 18:0, C 18:1, C 18:2, C 18:3, C 20:1, and C 22:1), protein content, and bioactive compounds such as glucosinolates and phytic acid. The level of genetic variation for the different variables, as depicted in the descriptive statistics tables, was found to be of various degrees, as shown by the coefficient of variation (CV), and these values highlighted the differences among each characteristic within the genotypes (14).

### 4.1 Spectral evaluation

**Figure 6:**
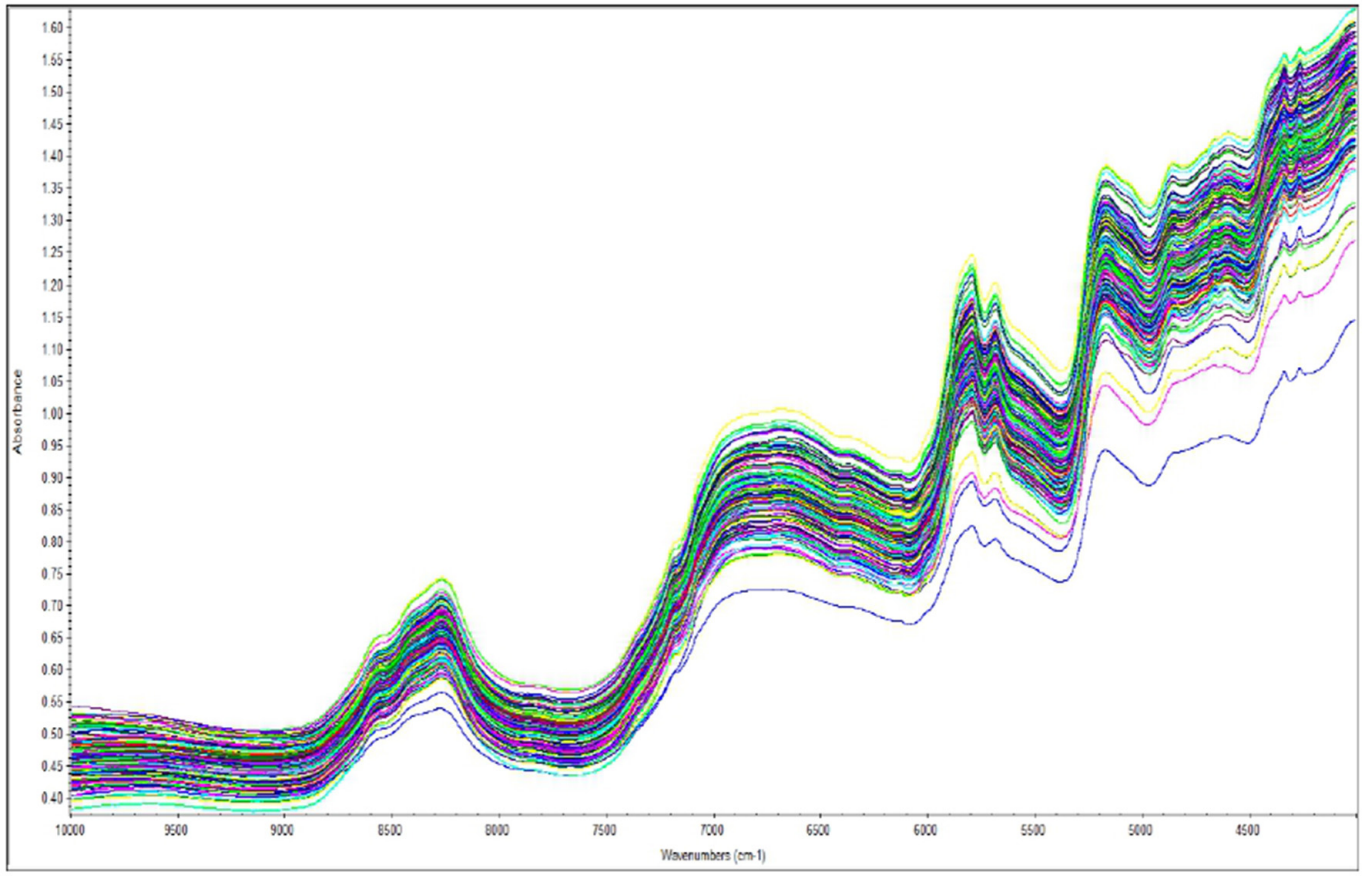
Raw NIR spectrum of samples at various wavenumbers (10,000–4,000 cm^−1^).

NIR spectral profiles of *Brassica* species are shown in Figure 6. The shape and rate of change in slope with wavelength represent the chemical information contained in each spectrum. The x-axis represents wavelength (cm^−1^), which corresponds to different vibrational frequencies of molecular bonds, while the y-axis shows absorbance intensity. These parameters provide a fairly adequate amount of accuracy for the projected models in relation to the reference values.

The overall spectrum shows strong absorption bands related to oil and water in this study. This study is consistent with findings from other oil seed crops such as *Brassica* sp., perilla, peanut, soybean, and sesame in the near-infrared region (17, 18). Moreover, as FT-NIR detection has enhanced spectral stability, all concerned bonds can substantially facilitate chemical bond analysis of biochemical attributes (19).

In our study, the NIR spectra indicate that water absorption bands are more prevalent due to the O-H bonds at approximately 9,500 cm^−1^, 7,500 cm^−1^, and 5,500 cm^−1^. According to the R^2^ analysis, the regions 5,700–5,800 cm^−1^ (C-H first overtones), 4,300–4,400 cm^−1^ (C-H, O-H combined band), and 8,200–8,300 cm^−1^ (C-H second overtones) contained crucial information for further determination of oil content and fatty acid profile. In the case of protein content and glucosinolates, significant relevance was found nearer to 6,500–6,600 cm^−1^ (N-H stretching initial overtone) and 4,800–4,900 cm^−1^ (C-H stretching and C=O combination) (20). The chemical bonds of phytic acid structure were mainly due to C-H (5,700–5,800 cm^−1^), P-OH, and O-H bonds (6,000–6,600 cm^−1^). Peaks observed at 5,000–7,600 cm^−1^ were associated with the combination bands of O-H and P-OH, respectively.

This study observed that the individual spectral data for each number of seed samples exhibited a lower coefficient of determination and RPD values below 2.85, which is considered very good for estimating biochemical compounds in seeds (20). Among the different seed samples from the mustard genotype that were used in this calibration, the desirable direction of traits revealed that sample no. 83, with spectrum title ST-82, recorded the highest actual oil content at 41.55% (Supplementary File 1). The coefficient of determination for oil content determination in mustard seed indicated excellent quantitative reliability (correlation coefficient of the FT- NIR predicted values calculated) against measured reference values (actual) was also noted, R^2^ = 0.92. Higher R^2^ values indicate greater reliability in NIR-based measurements (20).

### 4.2 Calibration model development and its evaluation

A calibration model was constructed to predict the oil content, fatty acid profile, glucosinolate content, phytic acid content, and protein content of the seed samples of mustard. The calibration analysis was carried out on the FT-NIR based on reflectance spectra and laboratory values and was assessed by TQ Analyst software with 64 scans. This analysis was then subjected to various scoring algorithms (modified partial least squares, partial least squares, and principal component regression) for making the calibration equation on the basis of Mahalanobis distance (H distance). H distance limits the spectral boundaries to remove outliers with samples H < 6 from the average spectrum. The F-ratio was suggested by Zhao et al. (21) as a better method for model development, particularly when the model will be used to predict future unknown samples.

The coefficient of determination (R^2^ = 0.9342) for oil content determination in *Brassica* seed indicated excellent quantitative reliability (22, 23). The correlation coefficient of FT- NIR predicted values (calculated) against measured reference values (actual) for oil concentration was also noted, R^2^ = 0.93, with RMSEC= 0.486 and RSMEP=0.540. Additionally, the RPD statistics for the equation of calibration were < 3 (2.85, 2.49), confirming its usefulness and accuracy for screening purposes (24). The PLS regression on FT-NIR data gives similar results for all parameters, for example, the coefficient of determination and the root mean square error in calibration (Table 1).

The oil content had the least deviation coefficient (CV = 2.1%), proving that the distribution of this trait was most homogeneous across genotypes. This finding also proved that FT-NIR spectroscopy is quite effective in assessing oil content with high precision, as shown by Smith et al. (25), who also obtained comparable levels of accuracy in their study on *Brassica napus*. On the other hand, erucic acid had the highest coefficient of variation (CV = 9.18%), indicating significant genetic differentiation for this trait. This study is in agreement with the study conducted by Sharafi et al. (26), where the authors noted that it is challenging to reduce the variation in erucic acid levels through breeding alone; however, they observed that specialized methods may help overcome this problem. Bianchi et al. (27) highlighted the importance of these steady characteristics for processing industries where oil quality consistency is a key issue.

The fatty acid profiles were of moderate to high heterogeneity. For example, the large CV values of oleic acid (CV = 7.50%) and linolenic acid (CV = 10.7%) proved that there are many genetic variations in the samples, and these results are in agreement with Li et al. (28), who also found high correlations between these fatty acids and erucic acid. The relationships that were identified in this study also support the genetic influences that determine these behaviors.

The variation in erucic acid was the most variable of the characteristics tested and may affect breeding strategies aimed at obtaining ideal fatty acid profiles containing reduced antinutritional components. The variation in linolenic acid, glucosinolates, phytic acid, and erucic acid (6, 29) indicates the possibility that there may be genetic remedies for stabilizing these constituents, with concomitant improvement of the fatty acid profile without sacrifice of protein or oil. Additionally, all attributes in this study are connected to some of these agronomic features, and our results may also influence breeders to select genotypes that maximize yield and nutritional value simultaneously (30).

Li et al. (28) quantified genetic variation in seed quality traits and discovered a significantly strong positive correlation between oleic acid and erucic acid. Our research was also able to demonstrate these relations, with a specific focus placed on the genetic relations that are essential in breeding strategies directed at enhancing such aspects. Our research also observed moderate to high variability of these fatty acids, which, in turn, supports their findings and confirms that these features are fairly sensitive to genetic variables. These results are in agreement with the study of Ghintala et al. (31), who also achieved variation in erucic acid content in genotypes. Our data also suggest that, with controlled breeding methods, it is possible to reduce this variability.

For this model presented, the errors in prediction are adequate, considering the standard deviation of the reference methods and the errors of the analytical determination obtained by the repeatability test. In fact, the RMSEP values should be lower than the standard deviation of the chemical determination chosen as reference data; moreover, the model errors are expected to be higher than the standard deviations obtained in repeatability conditions (8). The standard deviation is equal to 1.5%, while the analytical error is equal to 0.25% for oil content. For the protein content, the standard deviation is equal to 8.4%, while the error associated with the repeatability test is equal to 0.24% and has a similar pattern to all attributes (Table 1) (8).

Recent studies have supported the effectiveness of FT-NIR in evaluating the variability of all the attributes. For instance, Singh et al. (22) demonstrated that the FT-NIR can be reliably used to assess polyunsaturated fatty acids across various settings, achieving high prediction accuracy for the differentiation of the oleic and linolenic acid levels for different rapeseed cultivars. This finding is consistent with that of our study of the mild variation in oleic acid and confirms the reliability of the method across *Brassica* species.

Ammeter et al. (32) further found that FT-NIR spectroscopy may average the estimation of protein concentration, which was also observed in the present study. The small variations in protein content observed in our study predict that, although genetic differences do exist, FT-NIR spectroscopy can be relied upon for measuring it with a great deal of precision. Singh et al. (22) also observed the significance of FT-NIR to study the protein content of mustard seeds with a mild variation.

Genetic variation in glucosinolates and phytic acid also play a role in the breeding operation to produce crop varieties. Our findings were in agreement with the observations of Ali Redha et al. (33), who demonstrated that genetic diversity in glucosinolates and phytic acid is sufficient, which uses FT-NIR for selective breeding to add *Brassica* resistance to pests. The high CV in glucosinolates supports the versatility of the trait and highlights the importance of the FT-NIR to direct the breeding for lines and to simultaneously maximize the other characteristics. This study also confirms the reliability of FT-NIR in distinguishing differences in studied attributes among genotypes, so breeders can select quickly and with confidence.

### 4.3 Validation

The effectiveness of the equation was checked on the basis of standard errors of cross-validation (SEC), the coefficient of determination (R^2^), an estimate of R^2^, and standard error of cross-validation (SEP). The calibration results obtained for both the *Brassica* species showed high values of R^2^ for oil, protein, GSLs, C16:0, C18:1, C18:3, and C22:1, with a low value of SEC and SEP, except for C22:1, GSLs, which had a high value of SEP (Table 1). The results clearly indicate the heterogeneity obtained from validation of the equation (Tables 1, 2). Our results are in agreement with those reported by Singh et al. (22), who observed R^2^ values of the three species as 0.907, 0.922, 0.902 for oil, protein, and erucic acid content and SEP as 1.01, 0.68, 0.80 for oil content, protein content, and erucic acid content, respectively. Similarly, Prem et al. (34) also reported SECV 1.30, SEC 1.18, and R^2^ 0.94 for oil content and SECV = 12.19, R^2^ = 0.91, SEC = 2.18 for protein content. Petisco et al. (35) presented the calibration equations as more qualitative for seed oil content (R^2^ = 0.98, SEC = 0.90) and total glucosinolate content (R^2^ = 0.92, SEC = 8.19). They also studied the calibration statistics for the equations developed for fatty acids, including palmitic, stearic, oleic, linoleic, linolenic, and erucic acid, and our results are in agreement with these results. Ramesh et al. (20) also reported R^2^= 0.9972 and SEP= 0.0848 for intact seeds of castor, which is in accordance with our results. Validation of the developed calibration model was checked for accuracy, precision, and linearity. External validation results obtained in the case of *B. juncea, B. rapa, and B. napus* were reasonable for oil (0.920 and 0.934), phytic acid (0.749, 0.891), GSLs (0.986, 0.973), C18:1 (0.895, 0.858), C18:2 (0.867, 0.919), and C22:1 (0.898, 0.773) (Table 1), showing significant correlation between the predicted and laboratory FT-NIRS values (Figure 4). In contrast, correlation values between laboratory and predicted results were lower for C16:0 (0.996, 0.931), C18:0 (0.832, 0.885), C18:3 (0.911, 0.898), and C20:2 (0.834, 0.827) (Table 1). Our results were observed to be comparable with earlier studies for various biochemical parameters in oilseed *Brassica* species (36).

## 5 Conclusion

It could be summarized from the present investigation that FT-NIR is a highly accurate and powerful technique that could be utilized successfully for rapid mass screening in potential germplasm for selecting high oil, oleic acid, linoleic acid and protein, low glucosinolate, phytic acid, and erucic acid containing Indian mustard lines and thus to enhance the effectiveness of quality breeding program aimed at developing canola quality mustard. This regression model will be advanced by adding samples from different locations in India. In conclusion, the combination of simple devices with non-linear modeling may offer a very interesting and reliable tool for screening large numbers of samples for breeding programs.

## Data Availability

The original contributions presented in the study are included in the article/Supplementary material, further inquiries can be directed to the corresponding author.
