# Supplementary material for: Use of near-infrared spectroscopy for screening the oil content, protein, phytic acid, glucosinolates, and fatty acid profile in oilseed Brassica species
Source: Front Nutr. 2025 Sep 2;12:1632421. doi: 10.3389/fnut.2025.1632421 (PMC12439716; doi:10.3389/fnut.2025.1632421)
Supplement: Supplementary file 1 [file Data_Sheet_1.pdf]

Eicosenoic Acid   C:\RESULT Data\Workflows\Fatty acid eicosenoic 10-2-25.qnt
Revision: 12   Last saved on: Mon Feb 10 16:24:06 2025
Printed on: Tue Feb 11 10:20:22 2025

Calculated vs Actual Plot - Eicosenoic acid

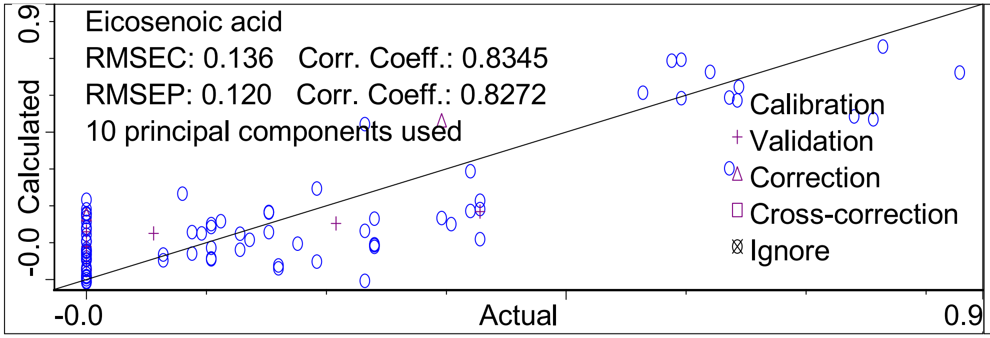

Difference vs Actual Plot - Eicosenoic acid

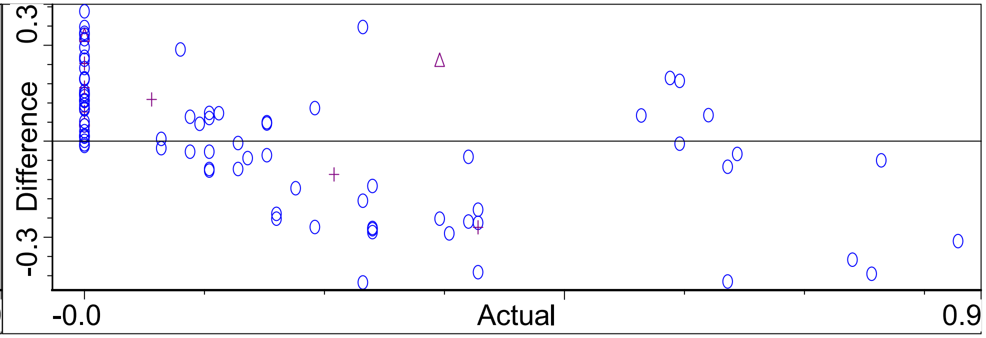

RMSEP: 0.120   Corr. Coeff.: 0.8272   10 principal components used
99.51% of variability described

Calibration Results Table - Eicosenoic acid

Index	File Name	Spectrum Title	Usage	Actual	Calculated	Diff. x Path
1	aicrp 2018 1.spa	Sample 2024-06-28 105808 GMT+0530	0	0.00	0.03	0.03
2	aicrp 2018 10 .spa	Sample 2024-06-28 151205 GMT+0530	1	0.00	0.06	0.06
3	aicrp 2018 11.spa	Sample 2024-06-28 151404 GMT+0530	0	0.00	0.10	0.10
4	aicrp 2018 12.spa	Sample 2024-06-28 151502 GMT+0530	0	0.00	0.15	0.15
5	aicrp 2018 13.spa	Sample 2024-06-28 151557 GMT+0530	0	0.00	0.21	0.21
7	aicrp 2018 15.spa	Sample 2024-06-28 151847 GMT+0530	0	0.37	0.21	-0.16
8	2017 1.spa	Sample 2024-07-01 101644 GMT+0530	0	0.61	0.74	0.13
9	2017 2.spa	Sample 2024-07-01 101731 GMT+0530	0	0.65	0.70	0.05
10	2017 3.spa	Sample 2024-07-01 101818 GMT+0530	0	0.00	0.09	0.09
11	2017 4.spa	Sample 2024-07-01 101909 GMT+0530	0	0.17	0.13	-0.04
12	2017 5.spa	Sample 2024-07-01 102150 GMT+0530	0	0.20	0.04	-0.16
13	2017 6.spa	Sample 2024-07-01 102238 GMT+0530	0	0.80	0.55	-0.25
14	2017 7.spa	Sample 2024-07-01 102324 GMT+0530	0	0.13	0.07	-0.06
15	2017 8.spa	Sample 2024-07-01 102410 GMT+0530	0	0.12	0.16	0.04
16	2017 9.spa	Sample 2024-07-01 102457 GMT+0530	0	0.14	0.20	0.06
17	2017 10.spa	Sample 2024-07-01 102555 GMT+0530	0	0.00	-0.01	-0.01
18	2017 13.spa	Sample 2024-07-01 102640 GMT+0530	0	0.58	0.63	0.05
19	2017 14.spa	Sample 2024-07-01 102730 GMT+0530	0	0.11	0.09	-0.02

20	2017 15.spa	Sample 2024-07-01 102812 GMT+0530	0	0.67	0.62	-0.05
21	aicrp 2023 6	Sample 2024-07-01 103633 GMT+0530	0	0.08	0.08	0.00
22	aicrp 2023 8.spa	Sample 2024-07-01 103829 GMT+0530	0	0.29	0.53	0.24
23	aicrp 2023 9	Sample 2024-07-01 103923 GMT+0530	0	0.00	0.13	0.13
24	aicrp 2023 11.spa	Sample 2024-07-01 104008 GMT+0530	0	0.19	0.23	0.04
25	aicrp 2023 10	Sample 2024-07-01 104201 GMT+0530	0	0.13	0.07	-0.06
26	aicrp 2023 4.spa	Sample 2024-07-01 104338 GMT+0530	0	0.24	0.06	-0.18
27	aicrp 2023 12.spa	Sample 2024-07-01 104434 GMT+0530	0	0.00	0.13	0.13
29	aicrp 2023 14.spa	Sample 2024-07-01 104627 GMT+0530	0	0.16	0.10	-0.06
30	aicrp 2023 2	Sample 2024-07-01 104720 GMT+0530	0	0.40	0.23	-0.17
33	aicrp 2023 1.spa	Sample 2024-07-01 105049 GMT+0530	0	0.00	-0.01	-0.01
34	aicrp 2023 18	Sample 2024-07-01 105209 GMT+0530	0	0.41	0.24	-0.17
36	aicrp 2023 19.spa	Sample 2024-07-01 105554 GMT+0530	0	0.13	0.18	0.05
37	aicrp 2023 7.spa	Sample 2024-07-01 105658 GMT+0530	0	0.00	0.20	0.20
38	aicrp 2023 16.spa	Sample 2024-07-01 105755 GMT+0530	2	0.00	0.22	0.22
40	aicrp 2023 9 r .s	Sample 2024-07-01 110402 GMT+0530	0	0.00	0.01	0.01
41	aicrp 2023 17 r .sp	Sample 2024-07-01 110546 GMT+0530	0	0.00	0.00	0.00
42	aicrp 2023 17 r sa	Sample 2024-07-01 110639 GMT+0530	0	0.00	0.09	0.09
43	aicrp 2023 1 r .sp	Sample 2024-07-01 110747 GMT+0530	0	0.00	0.04	0.04
44	aicrp 2023 25 r .sp	Sample 2024-07-01 110839 GMT+0530	0	0.00	0.22	0.22
45	aicrp 2023 22 r s	Sample 2024-07-01 110952 GMT+0530	0	0.62	0.75	0.13
46	aicrp 2023 19 r .s	Sample 2024-07-01 111050 GMT+0530	0	0.13	0.11	-0.02
47	aicrp 2023 2 r .s	Sample 2024-07-01 111201 GMT+0530	0	0.40	0.37	-0.03
48	aicrp 2023 6 r sa	Sample 2024-07-01 111251 GMT+0530	0	0.08	0.07	-0.01
49	aicrp 2023 14 r	Sample 2024-07-01 111339 GMT+0530	0	0.16	0.16	-0.00
50	aicrp 2023 24 r .s	Sample 2024-07-01 111438 GMT+0530	1	0.07	0.16	0.09
51	aicrp 2023 11 r.sp	Sample 2024-07-01 111523 GMT+0530	0	0.19	0.23	0.04
52	aicrp 2023 18 r .s	Sample 2024-07-01 111616 GMT+0530	0	0.41	0.27	-0.14
53	aicrp 2023 26 r .s	Sample 2024-07-01 111708 GMT+0530	0	0.83	0.79	-0.04
54	aicrp 2023 12 r .	Sample 2024-07-01 111809 GMT+0530	0	0.00	0.18	0.18
56	aicrp 2023 21 r .s	Sample 2024-07-01 111955 GMT+0530	0	0.00	0.27	0.27
58	aicrp 2023 16 r .s	Sample 2024-07-01 112521 GMT+0530	0	0.00	0.24	0.24
59	aicrp 2023 10 r .	Sample 2024-07-01 112629 GMT+0530	0	0.13	0.19	0.06

60	aicrp 2023 8 r .sSample 2024-07-01 112725 GMT+0530	0	0.29	0.17	-0.12
61	aicrp 2023 7 r .sSample 2024-07-01 112847 GMT+0530	0	0.00	0.10	0.10
62	aicrp 2023 5 r .sSample 2024-07-01 113001 GMT+0530	0	0.91	0.70	-0.21
63	aicrp 2023 4 r .sSample 2024-07-01 113054 GMT+0530	0	0.24	0.31	0.07
64	aicrp 2023 3 r .sSample 2024-07-01 113146 GMT+0530	0	0.67	0.38	-0.29
65	aicrp 2018 16.spa Sample 2024-06-28 152020 GMT+0530	0	0.00	0.01	0.01
66	aicrp 2018 17.spa Sample 2024-06-28 152204 GMT+0530	0	0.00	0.08	0.08
67	aicrp 2018 18.spa Sample 2024-06-28 152259 GMT+0530	0	0.00	0.08	0.08
68	aicrp 2018 19.spa Sample 2024-06-28 152439 GMT+0530	0	0.62	0.61	-0.01
69	aicrp 2018 20.spa Sample 2024-06-28 152639 GMT+0530	1	0.00	0.16	0.16
70	aicrp 2018 21.spa Sample 2024-06-28 152928 GMT+0530	0	0.00	0.01	0.01
71	aicrp 2018 22.spa Sample 2024-06-28 153019 GMT+0530	0	0.20	0.05	-0.15
72	aicrp 2021 1 sampSample 2024-06-28 102909 GMT+0530	0	0.10	0.29	0.19
73	aicrp 2021 2.spa Sample 2024-06-28 103249 GMT+0530	0	0.30	0.12	-0.18
74	aicrp 2021 3.spa Sample 2024-06-28 103439 GMT+0530	0	0.29	-0.00	-0.29
75	aicrp 2021 4.spa Sample 2024-06-28 103558 GMT+0530	0	0.11	0.16	0.05
77	aicrp 2021 6.spa Sample 2024-06-28 103810 GMT+0530	1	0.41	0.23	-0.18
79	aicrp 2021 8.spa Sample 2024-06-28 104016 GMT+0530	0	0.82	0.54	-0.28
80	aicrp 2021 9.spa Sample 2024-06-28 104111 GMT+0530	2	0.37	0.54	0.17
81	aicrp 2021 10.spa Sample 2024-06-28 104212 GMT+0530	0	0.19	0.16	-0.03
82	aicrp 2021 11.spa Sample 2024-06-28 104332 GMT+0530	0	0.38	0.19	-0.19
83	aicrp 2021 12.spa Sample 2024-06-28 104424 GMT+0530	0	0.30	0.11	-0.19
84	aicrp 2021 13.spa Sample 2024-06-28 104526 GMT+0530	0	0.30	0.21	-0.09
85	aicrp 2021 14.spa Sample 2024-06-28 104626 GMT+0530	0	0.41	0.14	-0.27
86	aicrp 2021 15.spa Sample 2024-06-28 104819 GMT+0530	1	0.26	0.19	-0.07
87	aicrp 2021 16.spa Sample 2024-06-28 104920 GMT+0530	0	0.30	0.12	-0.18
88	aicrp 2021 17.spa Sample 2024-06-28 105029 GMT+0530	0	0.22	0.12	-0.10
89	aicrp 2018 1.spa Sample 2024-06-28 105808 GMT+0530	0	0.00	0.03	0.03
90	aicrp 2018 2.spa Sample 2024-06-28 105951 GMT+0530	0	0.00	0.07	0.07
91	aicrp 2018 3.spa Sample 2024-06-28 110045 GMT+0530	0	0.00	0.07	0.07
92	aicrp 2018 4.spa Sample 2024-06-28 110154 GMT+0530	1	0.00	0.11	0.11
93	aicrp 2018 5.spa Sample 2024-06-28 110243 GMT+0530	0	0.00	0.10	0.10
94	aicrp 2018 6.spa Sample 2024-06-28 110352 GMT+0530	0	0.00	0.17	0.17

95	aicrp 2018 7.spa	Sample 2024-06-28 110435 GMT+0530	0	0.00	0.23	0.23
96	aicrp 2018 8.spa	Sample 2024-06-28 110521 GMT+0530	0	0.00	0.02	0.02
97	aicrp 2018 9.spa	Sample 2024-06-28 110943 GMT+0530	0	0.68	0.65	-0.03
6	aicrp 2018 14.spa	Sample 2024-06-28 151802 GMT+0530	3	0.00	-0.00	-0.00
28	aicrp 2023 3.spa	Sample 2024-07-01 104541 GMT+0530	3	0.67	-0.01	-0.68
31	aicrp 2023 13.spa	Sample 2024-07-01 104821 GMT+0530	3	0.70	0.01	-0.69
32	aicrp 2023 5.spa	Sample 2024-07-01 104912 GMT+0530	3	0.91	0.02	-0.89
35	aicrp 2023 17.spa	Sample 2024-07-01 105458 GMT+0530	3	0.00	0.58	0.58
39	aicrp 2023 20.spa	Sample 2024-07-01 105857 GMT+0530	3	1.31	-0.05	-1.36
55	aicrp 2023 23 r .:	Sample 2024-07-01 111901 GMT+0530	3	1.25	0.77	-0.48
57	aicrp 2023 20 r .	Sample 2024-07-01 112144 GMT+0530	3	1.31	0.82	-0.49
76	aicrp 2021 5.spa	Sample 2024-06-28 103658 GMT+0530	3	0.58	0.22	-0.36
78	aicrp 2021 7.spa	Sample 2024-06-28 103922 GMT+0530	3	1.86	0.76	-1.10
