# Supplementary material for: Use of near-infrared spectroscopy for screening the oil content, protein, phytic acid, glucosinolates, and fatty acid profile in oilseed Brassica species
Source: Front Nutr. 2025 Sep 2;12:1632421. doi: 10.3389/fnut.2025.1632421 (PMC12439716; doi:10.3389/fnut.2025.1632421)
Supplement: Supplementary file 2 [file Data_Sheet_2.pdf]

Mustard Seed - Erucic acid   C:\My Documents\Omnic\quant\Erucic Acid 7-3-25.qnt
Revision: 1   Last saved on: Fri Mar 07 13:10:53 2025
Printed on: Fri Mar 07 16:05:12 2025

Calculated vs Actual Plot - Erucic

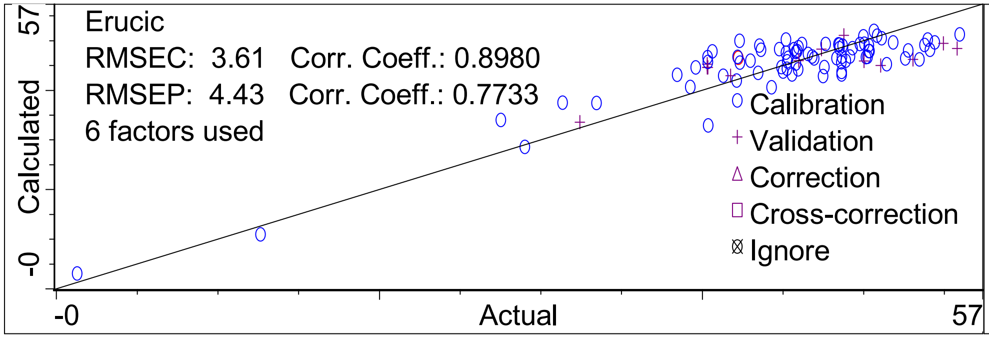

Difference vs Actual Plot - Erucic

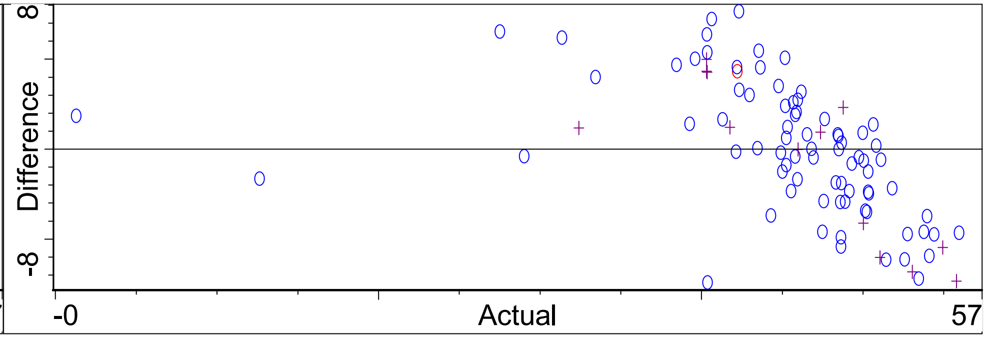

6 factors used

Calibration Results Table - Erucic

Index	File Name	Spectrum Title	Usage	Actual	Calculated	Diff. x Path
1	1 erucic sample.spa	42.22	0	42.22	46.55	4.33
2	2 erucic sample.spa	45.25	0	45.25	45.87	0.62
3	3 erucic sample.spa	45.87	0	45.87	47.94	2.07
4	4 erucic sample.spa	45.79	0	45.79	45.38	-0.41
5	5 erucic sample.spa	47.48	0	47.48	42.89	-4.59
6	6 erucic sample.spa	43.54	0	43.54	49.02	5.48
7	7 erucic sample.spa	42.13	0	42.13	41.99	-0.14
8	8 erucic sample.spa	40.31	1	40.31	45.30	4.99
9	9 erucic sample.spa	44.29	0	44.29	40.61	-3.68
10	10 erucic sample.sp	45.17	0	45.17	50.25	5.08
11	11 erucic sample.sp	46.18	0	46.18	49.37	3.19
12	12 erucic sample.sp	45.18	0	45.18	47.59	2.41
13	13 erucic sample.sp	42.19	0	42.19	46.75	4.56
14	14 erucic sample.sp	45.55	0	45.55	43.22	-2.33
15	15 erucic sample.sp	40.63	0	40.63	47.87	7.24
16	16 erucic sample.sp	43.64	0	43.64	48.18	4.54
17	17 erucic sample.sp	42.33	0	42.33	45.62	3.29
18	18 erucic sample.sp	39.60	0	39.60	44.63	5.03
19	19 erucic sample.sp	40.32	0	40.32	46.69	6.37

21	21 erucic sample.sp45.24	0	45.24	44.36	-0.88
22	22 erucic sample.sp43.45	0	43.45	43.51	0.06
23	23 erucic sample.sp41.30	0	41.30	42.96	1.66
24	24 erucic sample.sp48.63	0	48.63	43.73	-4.90
26	26 erucic sample.sp48.63	0	48.63	43.22	-5.41
27	27 erucic sample.sp45.93	0	45.93	44.26	-1.67
28	28 erucic sample.sp45.32	0	45.32	46.55	1.23
31	31 erucic sample.sp44.77	0	44.77	48.28	3.51
32	32 erucic sample.sp50.03	0	50.03	49.38	-0.65
33	33 erucic sample.sp45.96	0	45.96	48.70	2.74
34	34 erucic sample.sp42.97	0	42.97	45.98	3.01
35	35 erucic sample.sp44.90	0	44.90	44.70	-0.20
36	36 erucic sample.sp53.05	1	53.05	46.23	-6.82
37	37 erucic sample.sp48.77	1	48.77	51.08	2.31
38	38 erucic sample.sp48.48	0	48.48	49.21	0.73
39	39 erucic sample.sp40.31	1	40.31	44.65	4.34
41	41 erucic sample.sp45.79	0	45.79	47.70	1.91
42	42 erucic sample.sp50.24	0	50.24	46.76	-3.48
43	43 erucic sample.sp46.80	0	46.80	46.83	0.03
44	44 erucic sample.sp47.37	1	47.37	48.32	0.95
45	45 erucic sample.sp49.75	0	49.75	49.31	-0.44
46	46 erucic sample.sp50.13	0	50.13	46.71	-3.42
47	47 erucic sample.sp29.01	0	29.01	28.62	-0.39
48	48 erucic sample.sp32.41	1	32.41	33.59	1.18
49	49 erucic sample.sp27.52	0	27.52	34.07	6.55
50	50 erucic sample.sp40.37	0	40.37	32.96	-7.41
51	51 erucic sample.sp33.44	0	33.44	37.46	4.02
52	52 erucic sample.sp51.81	0	51.81	49.63	-2.18
53	53 erucic sample.sp41.76	1	41.76	42.97	1.21
54	54 erucic sample.sp48.60	0	48.60	45.65	-2.95
55	55 erucic sample.sp48.65	0	48.65	46.77	-1.88
56	64 erucic sample.sp46.54	0	46.54	47.36	0.82
57	56 erucic sample.sp45.01	0	45.01	43.76	-1.25

58	57 erucic sample.sp	50.01	1	50.01	45.89	-4.12
59	58 erucic sample.sp	45.97	1	45.97	45.96	-0.01
60	59 erucic sample.sp	55.79	1	55.79	48.45	-7.34
61	60 erucic sample.sp	47.56	0	47.56	44.68	-2.88
62	61 erucic sample.sp	53.44	0	53.44	46.24	-7.20
63	62 erucic sample.sp	47.63	0	47.63	49.31	1.68
64	63 erucic sample.sp	50.82	0	50.82	51.01	0.19
65	65 erucic sample.sp	40.35	1	40.35	44.63	4.28
66	66 erucic sample.sp	1.28	0	1.28	3.13	1.85
67	67 erucic sample.sp	48.68	0	48.68	49.04	0.36
68	68 erucic sample.sp	48.49	0	48.49	48.50	0.01
69	69 erucic sample.sp	45.68	0	45.68	48.29	2.61
70	70 erucic sample.sp	49.98	0	49.98	50.89	0.91
71	71 erucic sample.sp	55.94	0	55.94	51.30	-4.64
72	72 erucic sample.sp	50.63	0	50.63	52.00	1.37
74	74 erucic sample.sp	49.15	0	49.15	46.82	-2.33
77	77 erucic sample.sp	38.45	0	38.45	43.14	4.69
78	78 erucic sample.sp	50.31	0	50.31	49.06	-1.25
79	79 erucic sample.sp	49.31	0	49.31	48.52	-0.79
80	80 erucic sample.sp	51.04	1	51.04	45.02	-6.02
81	81 erucic sample.sp	51.11	0	51.11	50.52	-0.59
83	83 erucic sample.sp	48.43	0	48.43	49.24	0.81
85	85 erucic sample.sp	39.26	0	39.26	40.67	1.41
86	86 erucic sample.sp	54.39	0	54.39	49.65	-4.74
87	87 erucic sample.sp	52.57	0	52.57	46.44	-6.13
88	88 erucic sample.sp	54.10	0	54.10	48.17	-5.93
89	89 erucic sample.sp	48.31	0	48.31	46.47	-1.84
90	90 erucic sample.sp	31.35	0	31.35	37.55	6.20
91	91 erucic sample.sp	12.63	0	12.63	10.99	-1.64
92	92 erucic sample.sp	48.88	0	48.88	45.96	-2.92
93	93 erucic sample.sp	51.42	0	51.42	45.27	-6.15
94	94 erucic sample.sp	52.76	0	52.76	48.03	-4.73
95	95 erucic sample.sp	46.93	0	46.93	46.46	-0.47

96	96 erucic sample.sp:	50.31	0	50.31	47.94	-2.37
97	97 erucic sample.sp:	54.94	1	54.94	49.47	-5.47
99	99 erucic sample.sp:	40.34	0	40.34	45.72	5.38
100	100 erucic sample.s|	42.31	0	42.31	49.98	7.67
101	101 erucic sample.s|	50.35	0	50.35	47.89	-2.46
103	103 erucic sample.s|	53.96	0	53.96	50.24	-3.72
104	104 erucic sample.s|	53.77	0	53.77	49.17	-4.60
20	20 erucic sample.sp:	35.63	3	35.63	46.65	11.02
25	25 erucic sample.sp:	53.32	3	53.32	43.59	-9.73
29	29 erucic sample.sp:	54.33	3	54.33	46.68	-7.65
30	30 erucic sample.sp:	48.13	3	48.13	38.86	-9.27
40	40 erucic sample.sp:	35.41	3	35.41	45.47	10.06
73	73 erucic sample.sp:	14.28	3	14.28	45.88	31.60
75	75 erucic sample.sp:	56.95	3	56.95	47.98	-8.97
76	76 erucic sample.sp:	56.26	3	56.26	44.42	-11.84
82	82 erucic sample.sp:	55.26	3	55.26	45.57	-9.69
84	84 erucic sample.sp:	1.05	3	1.05	19.80	18.75
98	98 erucic sample.sp:	58.07	3	58.07	48.14	-9.93
102	102 erucic sample.s|	55.59	3	55.59	47.04	-8.55
