# Supplementary material for: Use of near-infrared spectroscopy for screening the oil content, protein, phytic acid, glucosinolates, and fatty acid profile in oilseed Brassica species
Source: Front Nutr. 2025 Sep 2;12:1632421. doi: 10.3389/fnut.2025.1632421 (PMC12439716; doi:10.3389/fnut.2025.1632421)
Supplement: Supplementary file 3 [file Data_Sheet_3.pdf]

mustard seed GSL   C:\My Documents\Omnic\quant\GSL 10 MARCH.qnt
Revision: 1   Last saved on: Mon Mar 10 12:28:03 2025
Printed on: Mon Mar 10 12:28:06 2025

Calculated vs Actual Plot - GSL

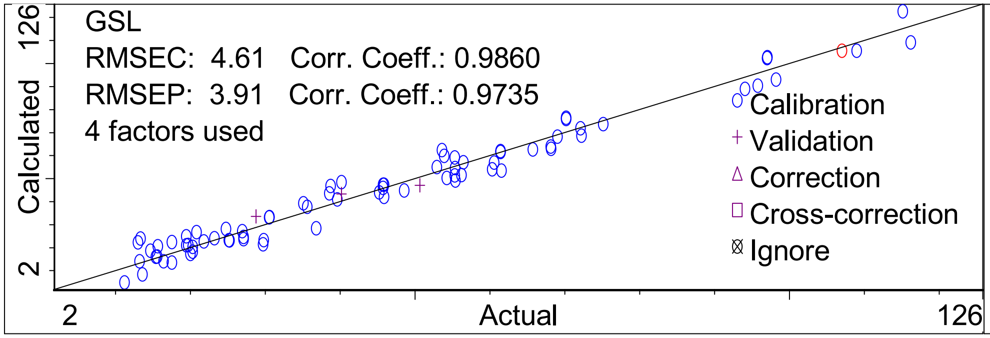

Difference vs Actual Plot - GSL

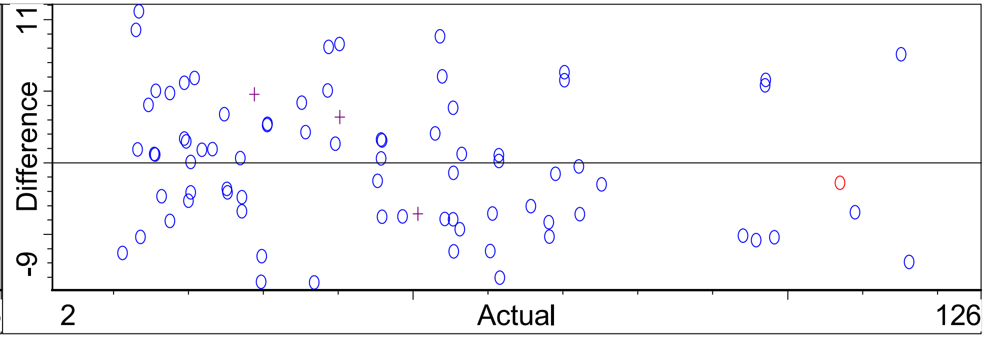

4 factors used

Calibration Results Table - GSL

Index	Spectrum Title	Usage	Actual	Calculated	Diff. x Path
1	Sample 2024-07-01 101644 GMT+0530	0	107.00	105.60	-1.40
2	Sample 2024-07-01 101731 GMT+0530	0	109.00	105.54	-3.46
3	Sample 2024-07-01 101818 GMT+0530	0	20.00	17.36	-2.64
4	Sample 2024-07-01 101909 GMT+0530	0	19.69	21.18	1.49
5	Sample 2024-07-01 102150 GMT+0530	0	72.24	68.65	-3.59
6	Sample 2024-07-01 102238 GMT+0530	0	115.16	122.75	7.59
7	Sample 2024-07-01 102324 GMT+0530	0	97.00	102.42	5.42
9	Sample 2024-07-01 102457 GMT+0530	0	116.20	109.27	-6.93
10	Sample 2024-07-01 102555 GMT+0530	0	15.62	20.66	5.04
11	Sample 2024-07-01 102640 GMT+0530	0	16.42	14.08	-2.34
13	Sample 2024-07-01 102812 GMT+0530	0	69.01	68.24	-0.77
16	Sample 2024-07-01 103923 GMT+0530	0	75.15	73.64	-1.51
18	Sample 2024-07-01 104201 GMT+0530	0	95.78	90.38	-5.40
19	Sample 2024-07-01 104338 GMT+0530	0	13.58	8.41	-5.17
20	Sample 2024-07-01 104434 GMT+0530	0	15.40	16.03	0.63
21	Sample 2024-07-01 104541 GMT+0530	0	68.11	63.96	-4.15
22	Sample 2024-07-01 104627 GMT+0530	0	60.29	54.12	-6.17
23	Sample 2024-07-01 104720 GMT+0530	0	11.18	4.87	-6.31
24	Sample 2024-07-01 104821 GMT+0530	0	55.41	49.22	-6.19

25	Sample 2024-07-01 104912 GMT+0530 0	25.20	23.13	-2.07
26	Sample 2024-07-01 105049 GMT+0530 0	13.19	14.13	0.94
27	Sample 2024-07-01 105209 GMT+0530 0	14.65	18.71	4.06
28	Sample 2024-07-01 105458 GMT+0530 0	72.11	71.86	-0.25
30	Sample 2024-07-01 105658 GMT+0530 0	26.91	27.24	0.33
31	Sample 2024-07-01 105755 GMT+0530 0	27.11	23.72	-3.39
32	Sample 2024-07-01 105857 GMT+0530 0	97.05	102.84	5.79
33	Sample 2024-07-01 110402 GMT+0530 0	68.17	63.02	-5.15
34	Sample 2024-07-01 110546 GMT+0530 0	19.43	25.02	5.59
36	Sample 2024-07-01 110747 GMT+0530 0	45.86	47.42	1.56
37	Sample 2024-07-01 110839 GMT+0530 0	61.42	61.55	0.13
38	Sample 2024-07-01 110952 GMT+0530 0	55.31	59.15	3.84
39	Sample 2024-07-01 111050 GMT+0530 0	45.73	47.36	1.63
40	Sample 2024-07-01 111201 GMT+0530 0	65.73	62.69	-3.04
41	Sample 2024-07-01 111251 GMT+0530 0	70.18	76.51	6.33
43	Sample 2024-07-01 111438 GMT+0530 0	25.14	23.32	-1.82
44	Sample 2024-07-01 111523 GMT+0530 0	27.11	24.72	-2.39
45	Sample 2024-07-01 111616 GMT+0530 0	94.05	88.96	-5.09
46	Sample 2024-07-01 111708 GMT+0530 0	19.43	21.13	1.70
47	Sample 2024-07-01 111809 GMT+0530 1	28.78	33.57	4.79
48	Sample 2024-07-01 111901 GMT+0530 0	45.86	42.10	-3.76
49	Sample 2024-07-01 111955 GMT+0530 0	61.42	61.94	0.52
50	Sample 2024-07-01 112144 GMT+0530 0	55.31	51.36	-3.95
51	Sample 2024-07-01 112521 GMT+0530 0	45.73	46.04	0.31
53	Sample 2024-07-01 112725 GMT+0530 0	53.89	59.92	6.03
54	Sample 2024-07-01 112847 GMT+0530 0	52.93	54.99	2.06
56	Sample 2024-07-01 113054 GMT+0530 0	70.18	75.94	5.76
57	Sample 2024-07-01 113146 GMT+0530 0	54.23	50.31	-3.92
59	Sample 2024-06-28 151404 GMT+0530 0	21.78	22.69	0.91
60	Sample 2024-06-28 151502 GMT+0530 1	50.64	47.08	-3.56
61	Sample 2024-06-28 151557 GMT+0530 0	40.17	48.48	8.31
62	Sample 2024-06-28 151802 GMT+0530 0	38.71	46.80	8.09
63	Sample 2024-06-28 151847 GMT+0530 0	20.82	26.75	5.93

65	Sample 2024-06-28 152204 GMT+0530 0	60.57	57.03	-3.54
66	Sample 2024-06-28 152259 GMT+0530 0	12.98	22.26	9.28
67	Sample 2024-06-28 152439 GMT+0530 0	24.76	28.17	3.41
68	Sample 2024-06-28 152639 GMT+0530 0	15.54	16.12	0.58
69	Sample 2024-06-28 152742 GMT+0530 0	13.35	23.94	10.59
70	Sample 2024-06-28 152928 GMT+0530 0	29.71	21.41	-8.30
72	Sample 2024-06-28 102909 GMT+0530 0	17.52	22.39	4.87
73	Sample 2024-06-28 103249 GMT+0530 0	56.46	57.08	0.62
74	Sample 2024-06-28 103439 GMT+0530 0	17.52	13.47	-4.05
75	Sample 2024-06-28 103558 GMT+0530 0	56.22	51.58	-4.64
76	Sample 2024-06-28 103658 GMT+0530 0	53.58	62.41	8.83
78	Sample 2024-06-28 103922 GMT+0530 0	23.19	24.15	0.96
79	Sample 2024-06-28 104016 GMT+0530 0	61.55	53.53	-8.02
80	Sample 2024-06-28 104111 GMT+0530 0	48.56	44.82	-3.74
82	Sample 2024-06-28 104332 GMT+0530 0	98.23	93.02	-5.21
83	Sample 2024-06-28 104424 GMT+0530 0	55.36	54.65	-0.71
84	Sample 2024-06-28 104526 GMT+0530 0	29.77	23.25	-6.52
85	Sample 2024-06-28 104626 GMT+0530 0	30.56	33.28	2.72
86	Sample 2024-06-28 104819 GMT+0530 0	38.56	43.62	5.06
87	Sample 2024-06-28 104920 GMT+0530 0	39.62	40.95	1.33
88	Sample 2024-06-28 105029 GMT+0530 0	45.23	43.98	-1.25
89	Sample 2024-06-28 105808 GMT+0530 0	20.31	18.25	-2.06
90	Sample 2024-06-28 105951 GMT+0530 0	30.51	33.15	2.64
91	Sample 2024-06-28 110045 GMT+0530 0	35.62	37.77	2.15
92	Sample 2024-06-28 110154 GMT+0530 1	40.19	43.39	3.20
93	Sample 2024-06-28 110243 GMT+0530 0	36.80	28.44	-8.36
96	Sample 2024-06-28 110521 GMT+0530 0	35.10	39.29	4.19
97	Sample 2024-06-28 110943 GMT+0530 0	20.30	20.36	0.06
8	Sample 2024-07-01 102410 GMT+0530 3	106.00	43.12	-62.88
12	Sample 2024-07-01 102730 GMT+0530 3	16.11	84.72	68.61
14	Sample 2024-07-01 103633 GMT+0530 3	80.04	61.12	-18.92
15	Sample 2024-07-01 103829 GMT+0530 3	76.64	36.76	-39.88
17	Sample 2024-07-01 104008 GMT+0530 3	16.25	28.50	12.25

29	Sample 2024-07-01 105554 GMT+0530 3	25.14	50.72	25.58
35	Sample 2024-07-01 110639 GMT+0530 3	28.78	65.88	37.10
42	Sample 2024-07-01 111339 GMT+0530 3	72.11	22.24	-49.87
52	Sample 2024-07-01 112629 GMT+0530 3	65.75	25.62	-40.13
55	Sample 2024-07-01 113001 GMT+0530 3	23.27	87.54	64.27
58	Sample 2024-06-28 151205 GMT+0530 3	52.91	15.89	-37.02
64	Sample 2024-06-28 152020 GMT+0530 3	61.71	22.30	-39.41
71	Sample 2024-06-28 153019 GMT+0530 3	57.86	46.95	-10.91
77	Sample 2024-06-28 103810 GMT+0530 3	15.78	3.95	-11.83
81	Sample 2024-06-28 104212 GMT+0530 3	105.23	40.00	-65.23
94	Sample 2024-06-28 110352 GMT+0530 3	28.90	48.11	19.21
95	Sample 2024-06-28 110435 GMT+0530 3	30.50	14.23	-16.27
