# Supplementary material for: Use of near-infrared spectroscopy for screening the oil content, protein, phytic acid, glucosinolates, and fatty acid profile in oilseed Brassica species
Source: Front Nutr. 2025 Sep 2;12:1632421. doi: 10.3389/fnut.2025.1632421 (PMC12439716; doi:10.3389/fnut.2025.1632421)
Supplement: Supplementary file 4 [file Data_Sheet_4.pdf]

Stearic acid C:\RESULT Data\Workflows\Fatty acid Linoleic 10-2-25.qnt
Revision: 3 Last saved on: Tue Feb 11 10:35:24 2025
Printed on: Tue Feb 11 10:35:28 2025

Calculated vs Actual Plot - Linoleic acid

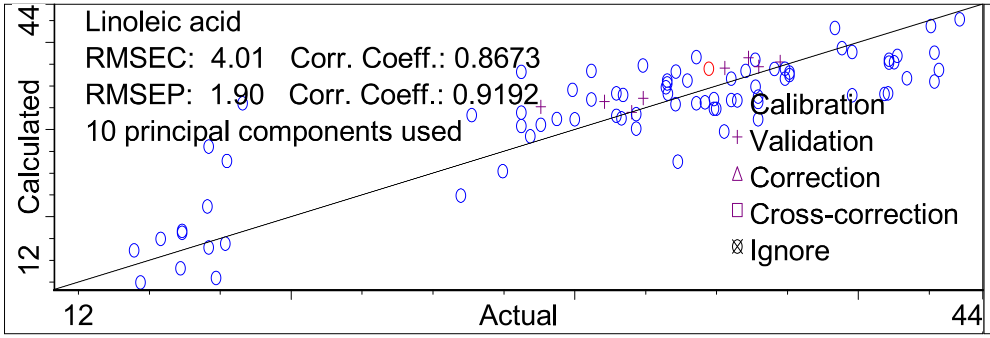

Difference vs Actual Plot - Linoleic acid

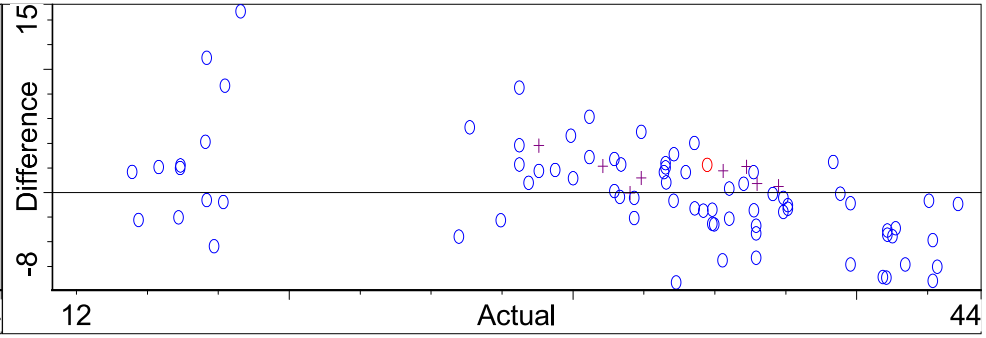

RMSEP: 1.90 Corr. Coeff.: 0.9192 10 principal components used
99.85% of variability described

Calibration Results Table - Linoleic acid

Index	File Name	Spectrum Title	Usage	Actual	Calculated	Diff. x Path
1	aicrp 2018 1.spa	Sample 2024-06-28 105808 GMT+0530	0	34.73	36.97	2.24
2	aicrp 2018 10 .spa	Sample 2024-06-28 151205 GMT+0530	1	32.01	31.97	-0.04
3	aicrp 2018 11.spa	Sample 2024-06-28 151404 GMT+0530	0	34.28	38.30	4.02
4	aicrp 2018 12.spa	Sample 2024-06-28 151502 GMT+0530	0	36.46	33.78	-2.68
5	aicrp 2018 13.spa	Sample 2024-06-28 151557 GMT+0530	0	36.37	34.93	-1.44
6	aicrp 2018 14.spa	Sample 2024-06-28 151802 GMT+0530	0	42.55	41.89	-0.66
7	aicrp 2018 15.spa	Sample 2024-06-28 151847 GMT+0530	0	27.46	25.20	-2.26
8	2017 1.spa	Sample 2024-07-01 101644 GMT+0530	0	16.16	18.37	2.21
9	2017 2.spa	Sample 2024-07-01 101731 GMT+0530	0	17.35	12.98	-4.37
10	2017 3.spa	Sample 2024-07-01 101818 GMT+0530	0	36.36	38.00	1.64
11	2017 4.spa	Sample 2024-07-01 101909 GMT+0530	0	37.04	36.93	-0.11
12	2017 5.spa	Sample 2024-07-01 102150 GMT+0530	0	18.29	33.01	14.72
14	2017 7.spa	Sample 2024-07-01 102324 GMT+0530	0	34.29	33.00	-1.29
16	2017 9.spa	Sample 2024-07-01 102457 GMT+0530	0	41.71	35.87	-5.84
19	2017 14.spa	Sample 2024-07-01 102730 GMT+0530	0	17.73	26.40	8.67
21	aicrp 2023 6	Sample 2024-07-01 103633 GMT+0530	0	42.68	35.51	-7.17
24	aicrp 2023 11.spa	Sample 2024-07-01 104008 GMT+0530	0	32.16	31.74	-0.42
25	aicrp 2023 10	Sample 2024-07-01 104201 GMT+0530	0	39.78	38.90	-0.88

26	aicrp 2023 4.spa	Sample 2024-07-01 104338 GMT+0530	0	33.63	26.33	-7.30
27	aicrp 2023 12.spa	Sample 2024-07-01 104434 GMT+0530	0	35.51	33.39	-2.12
28	aicrp 2023 3.spa	Sample 2024-07-01 104541 GMT+0530	0	30.58	36.74	6.16
29	aicrp 2023 14.spa	Sample 2024-07-01 104627 GMT+0530	0	34.91	33.51	-1.40
30	aicrp 2023 2	Sample 2024-07-01 104720 GMT+0530	0	28.11	31.96	3.85
31	aicrp 2023 13.spa	Sample 2024-07-01 104821 GMT+0530	0	26.36	31.65	5.29
34	aicrp 2023 18	Sample 2024-07-01 105209 GMT+0530	1	28.80	32.61	3.81
36	aicrp 2023 19.spa	Sample 2024-07-01 105554 GMT+0530	0	37.57	36.26	-1.31
37	aicrp 2023 7.spa	Sample 2024-07-01 105658 GMT+0530	0	31.46	31.59	0.13
38	aicrp 2023 16.spa	Sample 2024-07-01 105755 GMT+0530	0	36.46	31.17	-5.29
39	aicrp 2023 20.spa	Sample 2024-07-01 105857 GMT+0530	0	17.09	28.04	10.95
40	aicrp 2023 9 r .s	Sample 2024-07-01 110402 GMT+0530	0	40.92	34.07	-6.85
41	aicrp 2023 17 r .s	Sample 2024-07-01 110546 GMT+0530	0	41.08	37.67	-3.41
42	aicrp 2023 17 r s	Sample 2024-07-01 110639 GMT+0530	0	41.08	38.01	-3.07
43	aicrp 2023 1 r .s	Sample 2024-07-01 110747 GMT+0530	0	37.41	35.85	-1.56
44	aicrp 2023 25 r .sp	Sample 2024-07-01 110839 GMT+0530	0	35.27	29.77	-5.50
45	aicrp 2023 22 r	Sample 2024-07-01 110952 GMT+0530	0	17.68	16.90	-0.78
46	aicrp 2023 19 r .	Sample 2024-07-01 111050 GMT+0530	0	37.57	36.54	-1.03
47	aicrp 2023 2 r .s	Sample 2024-07-01 111201 GMT+0530	0	28.11	36.63	8.52
48	aicrp 2023 6 r s	Sample 2024-07-01 111251 GMT+0530	0	42.68	38.82	-3.86
49	aicrp 2023 14 r	Sample 2024-07-01 111339 GMT+0530	0	34.91	32.38	-2.53
50	aicrp 2023 24 r .s	Sample 2024-07-01 111438 GMT+0530	1	36.49	37.21	0.72
51	aicrp 2023 11 r.sp	Sample 2024-07-01 111523 GMT+0530	0	32.16	30.10	-2.06
52	aicrp 2023 18 r .	Sample 2024-07-01 111616 GMT+0530	0	28.80	30.55	1.75
53	aicrp 2023 26 r .	Sample 2024-07-01 111708 GMT+0530	0	16.15	18.15	2.00
54	aicrp 2023 12 r	.Sample 2024-07-01 111809 GMT+0530	0	35.51	35.85	0.34
55	aicrp 2023 23 r .	Sample 2024-07-01 111901 GMT+0530	0	17.05	21.18	4.13
56	aicrp 2023 21 r .s	Sample 2024-07-01 111955 GMT+0530	0	33.55	32.89	-0.66
57	aicrp 2023 20 r	.Sample 2024-07-01 112144 GMT+0530	0	17.09	16.47	-0.62
58	aicrp 2023 16 r .	Sample 2024-07-01 112521 GMT+0530	0	36.46	33.14	-3.32
59	aicrp 2023 10 r	.Sample 2024-07-01 112629 GMT+0530	0	39.78	33.93	-5.85
60	aicrp 2023 8 r .s	Sample 2024-07-01 112725 GMT+0530	0	34.98	32.40	-2.58
61	aicrp 2023 7 r .s	Sample 2024-07-01 112847 GMT+0530	0	31.46	34.17	2.71

62	aicrp 2023 5 r .sSample 2024-07-01 113001 GMT+0530	0	14.46	16.15	1.69
64	aicrp 2023 3 r .sSample 2024-07-01 113146 GMT+0530	0	30.58	33.47	2.89
65	aicrp 2018 16.spa Sample 2024-06-28 152020 GMT+0530	0	43.57	42.64	-0.93
66	aicrp 2018 17.spa Sample 2024-06-28 152204 GMT+0530	0	33.21	34.86	1.65
67	aicrp 2018 18.spa Sample 2024-06-28 152259 GMT+0530	0	34.59	33.13	-1.46
68	aicrp 2018 19.spa Sample 2024-06-28 152439 GMT+0530	0	16.10	14.08	-2.02
69	aicrp 2018 20.spa Sample 2024-06-28 152639 GMT+0530	1	37.24	37.75	0.51
70	aicrp 2018 21.spa Sample 2024-06-28 152928 GMT+0530	0	29.37	31.19	1.82
71	aicrp 2018 22.spa Sample 2024-06-28 153019 GMT+0530	0	31.70	33.98	2.28
72	aicrp 2021 1 sampSample 2024-06-28 102909 GMT+0530	0	32.40	37.34	4.94
73	aicrp 2021 2.spa Sample 2024-06-28 103249 GMT+0530	0	39.42	39.32	-0.10
74	aicrp 2021 3.spa Sample 2024-06-28 103439 GMT+0530	0	42.84	36.82	-6.02
75	aicrp 2021 4.spa Sample 2024-06-28 103558 GMT+0530	0	33.97	35.63	1.66
76	aicrp 2021 5.spa Sample 2024-06-28 103658 GMT+0530	0	31.65	31.29	-0.36
77	aicrp 2021 6.spa Sample 2024-06-28 103810 GMT+0530	1	35.29	37.06	1.77
78	aicrp 2021 7.spa Sample 2024-06-28 103922 GMT+0530	0	14.69	12.46	-2.23
79	aicrp 2021 8.spa Sample 2024-06-28 104016 GMT+0530	0	25.98	22.41	-3.57
80	aicrp 2021 9.spa Sample 2024-06-28 104111 GMT+0530	0	28.43	29.22	0.79
81	aicrp 2021 10.spa Sample 2024-06-28 104212 GMT+0530	0	29.92	34.55	4.63
82	aicrp 2021 11.spa Sample 2024-06-28 104332 GMT+0530	0	33.26	35.30	2.04
83	aicrp 2021 12.spa Sample 2024-06-28 104424 GMT+0530	0	41.26	37.70	-3.56
84	aicrp 2021 13.spa Sample 2024-06-28 104526 GMT+0530	0	33.56	36.66	3.10
85	aicrp 2021 14.spa Sample 2024-06-28 104626 GMT+0530	0	33.26	35.63	2.37
86	aicrp 2021 15.spa Sample 2024-06-28 104819 GMT+0530	1	36.12	38.23	2.11
87	aicrp 2021 16.spa Sample 2024-06-28 104920 GMT+0530	0	39.17	41.67	2.50
88	aicrp 2021 17.spa Sample 2024-06-28 105029 GMT+0530	0	30.00	31.17	1.17
89	aicrp 2018 1.spa Sample 2024-06-28 105808 GMT+0530	0	37.41	36.97	-0.44
90	aicrp 2018 2.spa Sample 2024-06-28 105951 GMT+0530	0	28.11	30.39	2.28
91	aicrp 2018 3.spa Sample 2024-06-28 110045 GMT+0530	0	33.28	34.11	0.83
92	aicrp 2018 4.spa Sample 2024-06-28 110154 GMT+0530	1	32.41	33.58	1.17
93	aicrp 2018 5.spa Sample 2024-06-28 110243 GMT+0530	0	36.02	36.73	0.71
94	aicrp 2018 6.spa Sample 2024-06-28 110352 GMT+0530	0	41.37	38.47	-2.90
95	aicrp 2018 7.spa Sample 2024-06-28 110435 GMT+0530	0	41.04	34.15	-6.89

Stearic acid   C:\RESULT Data\Workflows\Fatty acid Linoleic 10-2-25.qnt
Revision: 3   Last saved on: Tue Feb 11 10:35:24 2025
Printed on: Tue Feb 11 10:35:28 2025

96	aicrp 2018 8.spa	Sample 2024-06-28 110521 GMT+0530 1	31.05	33.19	2.14
97	aicrp 2018 9.spa	Sample 2024-06-28 110943 GMT+0530 0	15.40	17.46	2.06
13	2017 6.spa	Sample 2024-07-01 102238 GMT+0530 3	29.43	13.54	-15.89
15	2017 8.spa	Sample 2024-07-01 102410 GMT+0530 3	35.60	27.07	-8.53
17	2017 10.spa	Sample 2024-07-01 102555 GMT+0530 3	18.39	35.55	17.16
18	2017 13.spa	Sample 2024-07-01 102640 GMT+0530 3	31.82	15.74	-16.08
20	2017 15.spa	Sample 2024-07-01 102812 GMT+0530 3	33.27	19.91	-13.36
22	aicrp 2023 8.spa	Sample 2024-07-01 103829 GMT+0530 3	34.93	16.95	-17.98
23	aicrp 2023 9	Sample 2024-07-01 103923 GMT+0530 3	40.92	29.70	-11.22
32	aicrp 2023 5.spa	Sample 2024-07-01 104912 GMT+0530 3	14.46	31.16	16.70
33	aicrp 2023 1.spa	Sample 2024-07-01 105049 GMT+0530 3	37.41	28.50	-8.91
35	aicrp 2023 17.spa	Sample 2024-07-01 105458 GMT+0530 3	41.08	16.81	-24.27
63	aicrp 2023 4 r .s	Sample 2024-07-01 113054 GMT+0530 3	14.46	31.62	17.16
