# Supplementary material for: Use of near-infrared spectroscopy for screening the oil content, protein, phytic acid, glucosinolates, and fatty acid profile in oilseed Brassica species
Source: Front Nutr. 2025 Sep 2;12:1632421. doi: 10.3389/fnut.2025.1632421 (PMC12439716; doi:10.3389/fnut.2025.1632421)
Supplement: Supplementary file 5 [file Data_Sheet_5.pdf]

linolenic acid   C:\RESULT Data\Workflows\Fatty acid Linolenic 10-2-25.qnt
Revision: 3   Last saved on: Mon Feb 10 17:06:56 2025
Printed on: Mon Feb 10 17:07:05 2025

Calculated vs Actual Plot - Linolenic acid

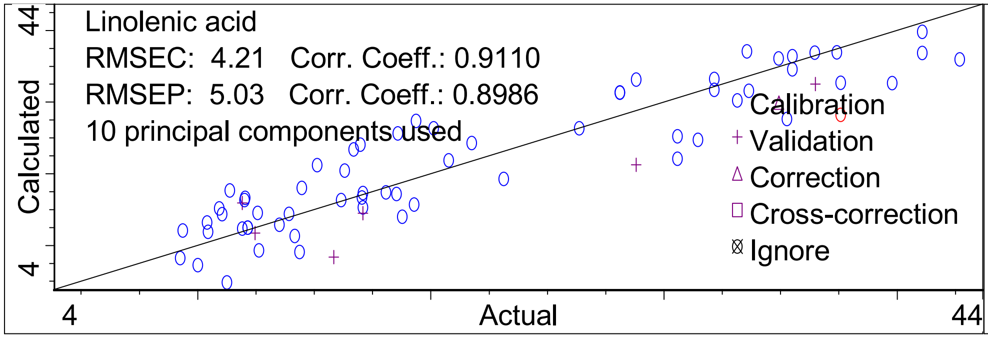

Difference vs Actual Plot - Linolenic acid

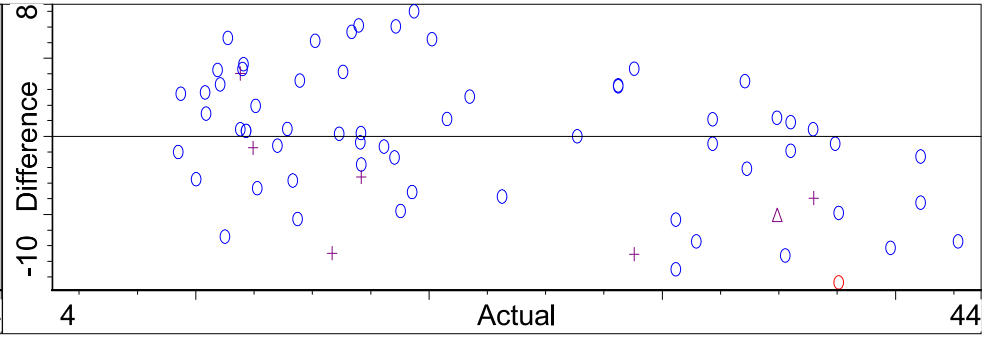

RMSEP: 5.03   Corr. Coeff.: 0.8986   10 principal components used
86.87% of variability described

Calibration Results Table - Linolenic acid

Index	File Name	Spectrum Title	Usage	Actual	Calculated	Diff. x Path
1	aicrp 2018 1.spa	Sample 2024-06-28 105808 GMT+0530	0	12.15	12.49	0.34
2	aicrp 2018 10 .spa	Sample 2024-06-28 151205 GMT+0530	1	12.46	11.73	-0.73
6	aicrp 2018 14.spa	Sample 2024-06-28 151802 GMT+0530	0	12.04	16.65	4.61
7	aicrp 2018 15.spa	Sample 2024-06-28 151847 GMT+0530	0	23.13	19.28	-3.85
8	2017 1.spa	Sample 2024-07-01 101644 GMT+0530	0	20.77	21.89	1.12
9	2017 2.spa	Sample 2024-07-01 101731 GMT+0530	0	21.75	24.30	2.55
12	2017 5.spa	Sample 2024-07-01 102150 GMT+0530	0	19.36	27.37	8.01
13	2017 6.spa	Sample 2024-07-01 102238 GMT+0530	0	19.28	15.71	-3.57
14	2017 7.spa	Sample 2024-07-01 102324 GMT+0530	0	18.58	25.63	7.05
15	2017 8.spa	Sample 2024-07-01 102410 GMT+0530	0	16.69	23.38	6.69
16	2017 9.spa	Sample 2024-07-01 102457 GMT+0530	0	20.12	26.33	6.21
18	2017 13.spa	Sample 2024-07-01 102640 GMT+0530	0	17.08	17.30	0.22
20	2017 15.spa	Sample 2024-07-01 102812 GMT+0530	0	18.78	14.00	-4.78
24	aicrp 2023 11.spa	Sample 2024-07-01 104008 GMT+0530	0	32.16	31.70	-0.46
25	aicrp 2023 10	Sample 2024-07-01 104201 GMT+0530	0	39.78	32.65	-7.13
27	aicrp 2023 12.spa	Sample 2024-07-01 104434 GMT+0530	0	35.51	36.42	0.91
28	aicrp 2023 3.spa	Sample 2024-07-01 104541 GMT+0530	0	30.58	22.08	-8.50
29	aicrp 2023 14.spa	Sample 2024-07-01 104627 GMT+0530	0	34.91	36.11	1.20

30	aicrp 2023 2	Sample 2024-07-01 104720 GMT+0530	0	28.11	31.31	3.20
31	aicrp 2023 13.spa	Sample 2024-07-01 104821 GMT+0530	0	26.36	26.36	0.00
32	aicrp 2023 5.spa	Sample 2024-07-01 104912 GMT+0530	0	14.46	18.04	3.58
34	aicrp 2023 18	Sample 2024-07-01 105209 GMT+0530	1	28.80	21.26	-7.54
36	aicrp 2023 19.spa	Sample 2024-07-01 105554 GMT+0530	0	37.57	28.22	-9.35
39	aicrp 2023 20.spa	Sample 2024-07-01 105857 GMT+0530	0	17.09	15.28	-1.81
41	aicrp 2023 17 r .s	Sample 2024-07-01 110546 GMT+0530	0	41.08	39.80	-1.28
42	aicrp 2023 17 r s	Sample 2024-07-01 110639 GMT+0530	0	41.08	36.84	-4.24
43	aicrp 2023 1 r .s	Sample 2024-07-01 110747 GMT+0530	0	37.41	36.95	-0.46
44	aicrp 2023 25 r .sp	Sample 2024-07-01 110839 GMT+0530	0	35.27	27.65	-7.62
46	aicrp 2023 19 r .	Sample 2024-07-01 111050 GMT+0530	0	37.57	32.68	-4.89
47	aicrp 2023 2 r .s	Sample 2024-07-01 111201 GMT+0530	0	28.11	31.37	3.26
48	aicrp 2023 6 r s	Sample 2024-07-01 111251 GMT+0530	0	42.68	35.97	-6.71
50	aicrp 2023 24 r .s	Sample 2024-07-01 111438 GMT+0530	1	36.49	32.53	-3.96
51	aicrp 2023 11 r.sp	Sample 2024-07-01 111523 GMT+0530	0	32.16	33.25	1.09
52	aicrp 2023 18 r .	Sample 2024-07-01 111616 GMT+0530	0	28.80	33.13	4.33
53	aicrp 2023 26 r .	Sample 2024-07-01 111708 GMT+0530	0	16.15	16.31	0.16
54	aicrp 2023 12 r	Sample 2024-07-01 111809 GMT+0530	0	35.51	34.59	-0.92
55	aicrp 2023 23 r .	Sample 2024-07-01 111901 GMT+0530	0	17.05	16.67	-0.38
56	aicrp 2023 21 r .s	Sample 2024-07-01 111955 GMT+0530	0	33.55	37.08	3.53
58	aicrp 2023 16 r .	Sample 2024-07-01 112521 GMT+0530	0	36.46	36.91	0.45
60	aicrp 2023 8 r .s	Sample 2024-07-01 112725 GMT+0530	2	34.93	29.91	-5.02
61	aicrp 2023 7 r .s	Sample 2024-07-01 112847 GMT+0530	0	31.46	24.73	-6.73
63	aicrp 2023 4 r .s	Sample 2024-07-01 113054 GMT+0530	0	33.63	31.57	-2.06
64	aicrp 2023 3 r .s	Sample 2024-07-01 113146 GMT+0530	0	30.58	25.24	-5.34
65	aicrp 2018 16.spa	Sample 2024-06-28 152020 GMT+0530	0	10.01	7.27	-2.74
66	aicrp 2018 17.spa	Sample 2024-06-28 152204 GMT+0530	0	12.57	14.52	1.95
67	aicrp 2018 18.spa	Sample 2024-06-28 152259 GMT+0530	0	12.63	9.30	-3.33
68	aicrp 2018 19.spa	Sample 2024-06-28 152439 GMT+0530	0	18.52	17.17	-1.35
70	aicrp 2018 21.spa	Sample 2024-06-28 152928 GMT+0530	0	14.37	9.07	-5.30
71	aicrp 2018 22.spa	Sample 2024-06-28 153019 GMT+0530	0	16.98	24.08	7.10
72	aicrp 2021 1 samp	Sample 2024-06-28 102909 GMT+0530	0	13.50	12.90	-0.60
73	aicrp 2021 2.spa	Sample 2024-06-28 103249 GMT+0530	0	11.38	17.69	6.31

74	aicrp 2021 3.spa	Sample 2024-06-28 103439 GMT+0530	0	11.05	14.37	3.32
75	aicrp 2021 4.spa	Sample 2024-06-28 103558 GMT+0530	0	11.91	12.36	0.45
77	aicrp 2021 6.spa	Sample 2024-06-28 103810 GMT+0530	1	15.84	8.36	-7.48
80	aicrp 2021 9.spa	Sample 2024-06-28 104111 GMT+0530	0	13.92	14.40	0.48
81	aicrp 2021 10.spa	Sample 2024-06-28 104212 GMT+0530	0	18.07	17.42	-0.65
82	aicrp 2021 11.spa	Sample 2024-06-28 104332 GMT+0530	0	15.12	21.23	6.11
83	aicrp 2021 12.spa	Sample 2024-06-28 104424 GMT+0530	0	10.40	13.22	2.82
84	aicrp 2021 13.spa	Sample 2024-06-28 104526 GMT+0530	0	10.93	15.18	4.25
85	aicrp 2021 14.spa	Sample 2024-06-28 104626 GMT+0530	0	16.31	20.43	4.12
86	aicrp 2021 15.spa	Sample 2024-06-28 104819 GMT+0530	1	11.91	15.94	4.03
88	aicrp 2021 17.spa	Sample 2024-06-28 105029 GMT+0530	0	9.36	12.10	2.74
89	aicrp 2018 1.spa	Sample 2024-06-28 105808 GMT+0530	0	12.15	12.49	0.34
90	aicrp 2018 2.spa	Sample 2024-06-28 105951 GMT+0530	0	12.01	16.32	4.31
91	aicrp 2018 3.spa	Sample 2024-06-28 110045 GMT+0530	0	14.16	11.34	-2.82
92	aicrp 2018 4.spa	Sample 2024-06-28 110154 GMT+0530	1	17.09	14.48	-2.61
93	aicrp 2018 5.spa	Sample 2024-06-28 110243 GMT+0530	0	10.44	11.90	1.46
94	aicrp 2018 6.spa	Sample 2024-06-28 110352 GMT+0530	0	11.25	4.83	-6.42
95	aicrp 2018 7.spa	Sample 2024-06-28 110435 GMT+0530	0	9.25	8.25	-1.00
3	aicrp 2018 11.spa	Sample 2024-06-28 151404 GMT+0530	3	10.80	20.07	9.27
4	aicrp 2018 12.spa	Sample 2024-06-28 151502 GMT+0530	3	10.64	23.25	12.61
5	aicrp 2018 13.spa	Sample 2024-06-28 151557 GMT+0530	3	13.65	24.76	11.11
10	2017 3.spa	Sample 2024-07-01 101818 GMT+0530	3	6.80	33.77	26.97
11	2017 4.spa	Sample 2024-07-01 101909 GMT+0530	3	7.67	37.16	29.49
17	2017 10.spa	Sample 2024-07-01 102555 GMT+0530	3	13.53	33.24	19.71
19	2017 14.spa	Sample 2024-07-01 102730 GMT+0530	3	9.74	31.95	22.21
21	aicrp 2023 6	Sample 2024-07-01 103633 GMT+0530	3	12.15	32.24	20.09
22	aicrp 2023 8.spa	Sample 2024-07-01 103829 GMT+0530	3	3.23	18.81	15.58
23	aicrp 2023 9	Sample 2024-07-01 103923 GMT+0530	3	40.52	26.26	-14.26
26	aicrp 2023 4.spa	Sample 2024-07-01 104338 GMT+0530	3	33.68	22.12	-11.56
33	aicrp 2023 1.spa	Sample 2024-07-01 105049 GMT+0530	3	37.41	13.62	-23.79
35	aicrp 2023 17.spa	Sample 2024-07-01 105458 GMT+0530	3	41.08	5.74	-35.34
37	aicrp 2023 7.spa	Sample 2024-07-01 105658 GMT+0530	3	31.46	17.73	-13.73
38	aicrp 2023 16.spa	Sample 2024-07-01 105755 GMT+0530	3	36.46	23.66	-12.80

linolenic acid   C:\RESULT Data\Workflows\Fatty acid Linolenic 10-2-25.qnt
Revision: 3   Last saved on: Mon Feb 10 17:06:56 2025
Printed on: Mon Feb 10 17:07:05 2025

40	aicrp 2023	9 r .s	Sample 2024-07-01 110402 GMT+0530 3	40.92	29.02	-11.90
45	aicrp 2023	22 r :	Sample 2024-07-01 110952 GMT+0530 3	17.68	32.73	15.05
49	aicrp 2023	14 r	Sample 2024-07-01 111339 GMT+0530 3	34.91	22.59	-12.32
57	aicrp 2023	20 r .	Sample 2024-07-01 112144 GMT+0530 3	17.09	28.84	11.75
59	aicrp 2023	10 r .	Sample 2024-07-01 112629 GMT+0530 3	39.78	26.29	-13.49
62	aicrp 2023	5 r .s	Sample 2024-07-01 113001 GMT+0530 3	14.46	34.26	19.80
69	aicrp 2018	20.spa	Sample 2024-06-28 152639 GMT+0530 3	60.51	8.45	-52.06
76	aicrp 2021	5.spa	Sample 2024-06-28 103658 GMT+0530 3	15.22	5.00	-10.22
78	aicrp 2021	7.spa	Sample 2024-06-28 103922 GMT+0530 3	21.24	-10.21	-31.45
79	aicrp 2021	8.spa	Sample 2024-06-28 104016 GMT+0530 3	18.88	6.79	-12.09
87	aicrp 2021	16.spa	Sample 2024-06-28 104920 GMT+0530 3	12.11	25.73	13.62
96	aicrp 2018	8.spa	Sample 2024-06-28 110521 GMT+0530 3	14.21	1.92	-12.29
97	aicrp 2018	9.spa	Sample 2024-06-28 110943 GMT+0530 3	18.51	-1.40	-19.91
