# Supplementary material for: Use of near-infrared spectroscopy for screening the oil content, protein, phytic acid, glucosinolates, and fatty acid profile in oilseed Brassica species
Source: Front Nutr. 2025 Sep 2;12:1632421. doi: 10.3389/fnut.2025.1632421 (PMC12439716; doi:10.3389/fnut.2025.1632421)
Supplement: Supplementary file 6 [file Data_Sheet_6.pdf]

Muster seed oil content   C:\My Documents\Omnic\quant\Mustard Seed - oil content14.qnt
Revision: 3   Last saved on: Wed Mar 13 11:28:02 2024
Printed on: Fri Mar 07 14:20:14 2025

Calculated vs Actual Plot - Oil

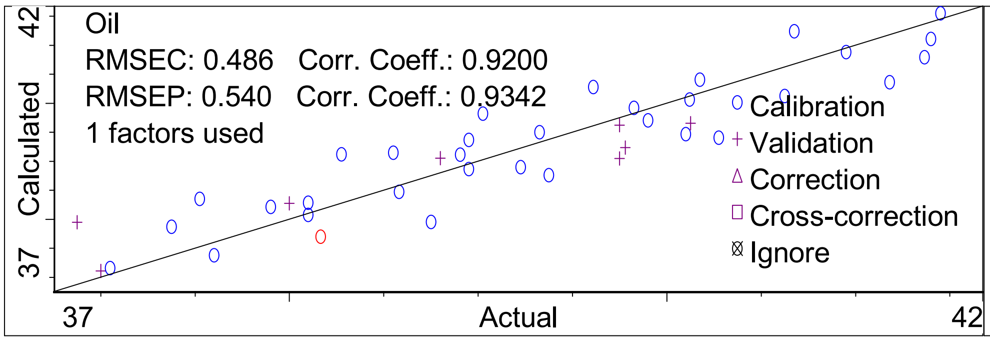

Difference vs Actual Plot - Oil

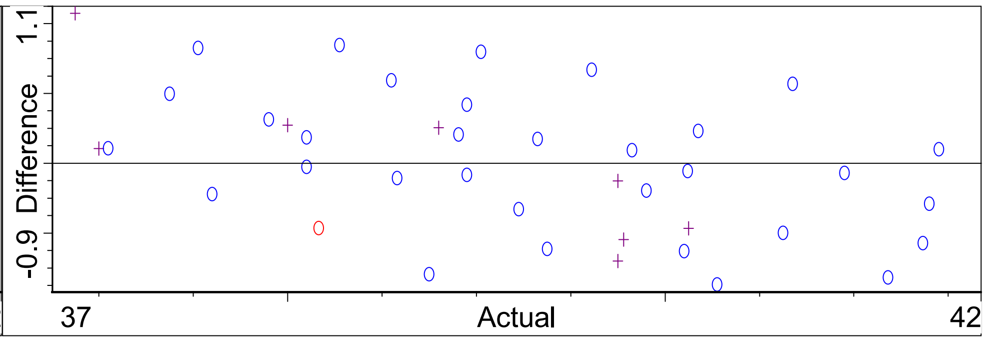

1 factors used

Calibration Results Table - Oil

Index	File Name	Spectrum Title	Usage	Actual	Calculated	Diff. x Path
5	drmr-5 germplasm s	38.17	0	38.17	37.70	-0.46
8	drmr-8 germplasm s	39.22	0	39.22	38.90	-0.33
11	drmr-11 germplasm	38.75	0	38.75	37.96	-0.79
16	drmr-16 germplasm	36.88	1	36.88	37.95	1.07
18	drmr-18 germplasm	37.60	0	37.60	37.38	-0.22
23	drmr-23 germplasm	40.10	0	40.10	39.47	-0.63
24	drmr-24 germplasm	37.00	1	37.00	37.11	0.11
28	drmr-28 germplasm	37.05	0	37.05	37.16	0.11
35	drmr-35 germplasm	37.90	0	37.90	38.21	0.31
38	drmr-.38 germplasm	37.38	0	37.38	37.87	0.50
39	drmr-39 germplasm	39.61	0	39.61	40.28	0.67
41	41 anubhuti sharma	40.95	0	40.95	40.88	-0.07
42	42 anubhuti sharma	40.28	0	40.28	39.41	-0.87
45	45 anubhuti sharma	37.53	0	37.53	38.35	0.83
46	46 anubhuti sharma	38.55	0	38.55	39.14	0.59
47	47 anubhuti sharma	38.10	0	38.10	38.08	-0.02
48	48 anubhuti sharma	39.75	1	39.75	39.62	-0.13
57	57 anubhuti sharma	38.28	0	38.28	39.12	0.85
59	59 anubhuti sharma	38.95	0	38.95	38.87	-0.08

60	60 anubhuti sharma 40.12	1	40.13	39.66	-0.47
62	62 anubhuti ma'am t39.38	0	39.38	38.76	-0.61
68	68 anubhuti ma'am t38.10	0	38.10	38.29	0.19
69	69 anubhuti ma'am t38.00	1	38.00	38.27	0.27
72	71 anubhuti ma'am t39.33	0	39.33	39.50	0.17
73	72 anubhuti ma'am t38.80	1	38.80	39.06	0.26
75	74 anubhuti ma'am t39.83	0	39.83	39.92	0.09
76	75 anubhuti ma'am t39.90	0	39.90	39.70	-0.20
77	76 anubhuti ma'am t40.62	0	40.63	40.13	-0.50
78	77 anubhuti ma'am t39.75	1	39.75	39.05	-0.70
79	78 anubhuti ma'am t39.03	0	39.03	39.82	0.80
81	80 anubhuti ma'am t41.40	0	41.40	41.11	-0.29
82	81 anubhuti sharma 41.18	0	41.18	40.36	-0.82
83	82 anubhuti sharma 41.45	0	41.45	41.55	0.10
84	83 anubhuti sharma 40.17	0	40.17	40.41	0.23
86	85 anubhuti sharma 40.67	0	40.67	41.24	0.57
88	87 anubhuti sharma 41.37	0	41.37	40.79	-0.57
90	89 anubhuti sharma 38.95	0	38.95	39.37	0.42
94	94 anubhuti sharma 40.12	0	40.12	40.07	-0.05
98	98 anubhuti sharma 38.58	0	38.58	38.47	-0.11
99	99 anubhuti sharma 38.90	0	38.90	39.11	0.21
100	100 anubhuti sharm39.78	1	39.78	39.23	-0.55
1	drmr-1 germplasm s36.15	3	36.15	37.74	1.59
2	drmr-2 germplasm s40.35	3	40.35	38.50	-1.85
3	drmr-3 germplasm s41.22	3	41.22	38.88	-2.35
4	drmr-4 germplasm s41.65	3	41.65	41.01	-0.64
6	drmr-6 germplasm s40.85	3	40.85	38.00	-2.85
7	drmr-7 germplasm s41.75	3	41.75	39.27	-2.48
9	drmr-9 germplasm s38.03	3	38.03	39.80	1.77
10	drmr-10 germplasm 38.55	3	38.55	36.94	-1.61
12	drmr-12 germplasm 41.97	3	41.97	39.64	-2.34
13	drmr-13 germplasm 30.27	3	30.28	36.10	5.83
14	drmr-14 germplasm 36.83	3	36.83	38.18	1.35

15	drmr-15 germplasm 40.37	3	40.37	38.61	-1.76
17	drmr-17 germplasm 36.62	3	36.63	38.75	2.13
19	drmr-19 germplasm 40.45	3	40.45	38.65	-1.80
20	drmr-20 germplasm 40.88	3	40.88	38.40	-2.48
21	drmr-21 germplasm 41.22	3	41.22	39.17	-2.06
22	drmr-22 germplasm 38.22	3	38.22	39.31	1.09
25	drmr-25 germplasm 40.10	3	40.10	38.06	-2.04
26	drmr-26 germplasm 39.28	3	39.28	37.91	-1.36
27	drmr-27 germplasm 39.15	3	39.15	38.02	-1.13
29	drmr-29 germplasm 40.45	3	40.45	39.36	-1.09
30	drmr-30 germplasm 41.28	3	41.28	38.19	-3.09
31	drmr-31 germplasm 39.60	3	39.60	38.52	-1.08
32	drmr-32 germplasm 36.58	3	36.58	38.98	2.40
33	drmr-33 germplasm 38.53	3	38.53	37.24	-1.29
34	drmr-34 germplasm 36.90	3	36.90	37.35	0.45
36	drmr-36 germplasm 36.95	3	36.95	38.14	1.19
37	drmr-37 germplasm 39.72	3	39.72	38.37	-1.35
40	drmr-40 germplasm 41.58	3	41.58	40.47	-1.10
43	43 anubhuti sharma 40.05	3	40.05	39.62	-0.43
44	44 anubhuti sharma 38.62	3	38.63	38.59	-0.03
49	49 anubhuti sharma 40.12	3	40.13	39.39	-0.73
50	50 anubhuti sharma 36.75	3	36.75	39.35	2.60
51	51 anubhuti sharma 35.00	3	35.00	38.95	3.95
52	52 anubhuti sharma 37.53	3	37.53	38.62	1.09
53	53 anubhuti sharma 40.03	3	40.03	40.07	0.04
54	54 anubhuti sharma 37.72	3	37.72	39.41	1.68
55	55 anubhuti sharma 37.72	3	37.72	39.10	1.38
56	56 anubhuti sharma 39.10	3	39.10	39.52	0.42
58	58 anubhuti sharma 38.22	3	38.22	39.40	1.18
61	61 anubhuti ma'am t40.62	3	40.63	39.54	-1.08
63	63 anubhuti ma'am t40.28	3	40.28	39.29	-0.99
64	64 anubhuti ma'am t36.25	3	36.25	37.93	1.68
65	65 anubhuti ma'am t40.97	3	40.97	39.40	-1.58

66	66 anubhuti ma'am t40.60	3	40.60	39.59	-1.01
67	67 anubhuti ma'am t40.50	3	40.50	39.27	-1.23
70	70 anubhuti ma'am t36.08	3	36.08	38.96	2.88
71	70 repet anubhuti m40.38	3	40.38	39.36	-1.01
74	73 anubhuti ma'am t40.13	3	40.13	38.39	-1.74
80	79 anubhuti ma'am t41.97	3	41.97	39.62	-2.35
85	84 anubhuti sharma 40.40	3	40.40	41.51	1.11
87	86 anubhuti sharma 36.78	3	36.78	41.02	4.24
89	88 anubhuti sharma 40.76	3	40.76	40.31	-0.45
91	90 anubhuti sharma 37.04	3	37.04	39.46	2.41
92	91 anubhuti sharma 37.62	3	37.63	41.04	3.42
93	93 anubhuti sharma 37.42	3	37.42	39.42	2.00
95	95 anubhuti sharma 35.91	3	35.91	40.62	4.71
96	96 anubhuti sharma 36.83	3	36.83	39.76	2.93
97	97 anubhuti sharma 36.55	3	36.55	39.83	3.28
