# Supplementary material for: Use of near-infrared spectroscopy for screening the oil content, protein, phytic acid, glucosinolates, and fatty acid profile in oilseed Brassica species
Source: Front Nutr. 2025 Sep 2;12:1632421. doi: 10.3389/fnut.2025.1632421 (PMC12439716; doi:10.3389/fnut.2025.1632421)
Supplement: Supplementary file 7 [file Data_Sheet_7.pdf]

C:\RESULT Data\Workflows\Fatty acid table Oleic ACID 7-03-25.qnt
Revision: 1 Last saved on: Fri Mar 07 14:00:48 2025
Printed on: Fri Mar 07 14:00:52 2025

Calculated vs Actual Plot - Oleic

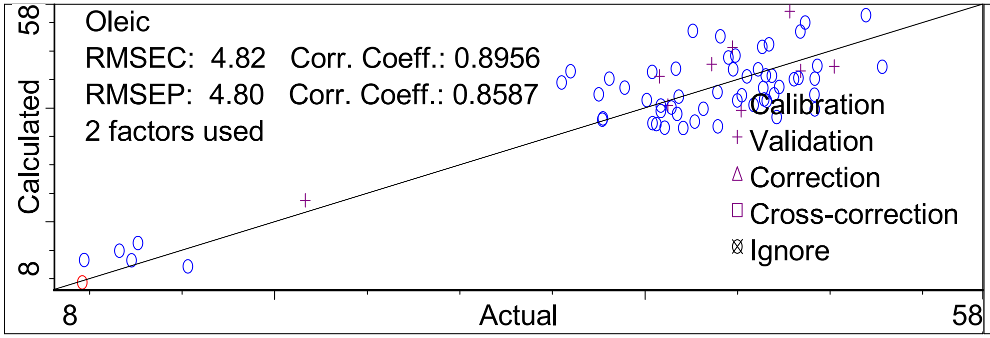

Difference vs Actual Plot - Oleic

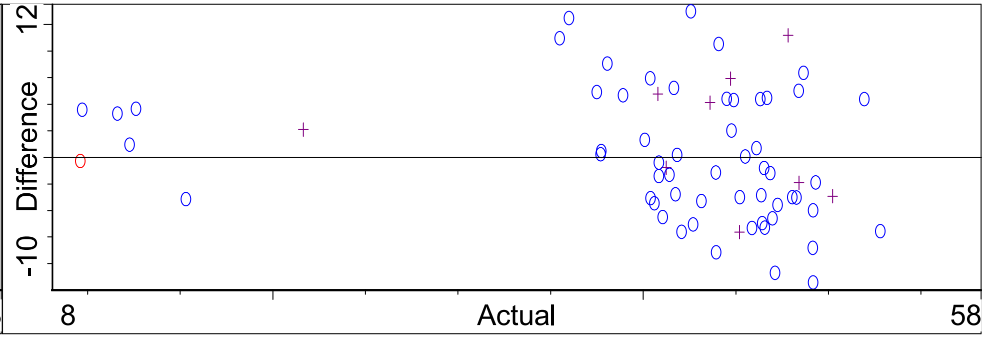

2 factors used

Calibration Results Table - Oleic

Index	File Name	Spectrum Title	Usage	Actual	Calculated	Diff. x Path
2	2017 2.spa	9.60	0	9.60	9.33	-0.27
4	2017 4.spa	46.33	0	46.33	50.71	4.38
5	2017 5.spa	35.98	0	35.98	46.46	10.48
7	2017 7.spa	43.15	0	43.15	39.85	-3.30
8	2017 8.spa	41.83	0	41.83	42.01	0.18
9	2017 9.spa	35.49	0	35.49	44.46	8.97
10	2017 10.spa	37.50	0	37.50	42.40	4.90
11	2017 13.spa	21.65	1	21.65	23.74	2.09
12	2017 14.spa	46.98	0	46.98	42.38	-4.60
13	2017 15.spa	15.30	0	15.30	12.16	-3.14
14	aicrp 2018 1.spa	46.38	0	46.38	43.52	-2.86
15	aicrp 2018 2.spa	48.41	1	48.41	46.50	-1.91
17	aicrp 2018 4.spa	44.07	0	44.07	52.60	8.53
18	aicrp 2018 5.spa	46.43	0	46.43	41.47	-4.96
19	aicrp 2018 6.spa	41.24	1	41.24	40.45	-0.79
20	aicrp 2018 7.spa	42.69	0	42.69	37.64	-5.05
21	aicrp 2018 8.spa	46.57	0	46.57	41.27	-5.30
22	aicrp 2018 9.spa	9.71	0	9.71	13.28	3.57
23	aicrp 2018 10 .spa	50.22	1	50.22	47.31	-2.91

24	aicrp 2018 11.spa	49.31	0	49.31	47.41	-1.90
25	aicrp 2018 12.spa	46.69	0	46.69	51.17	4.48
26	aicrp 2018 13.spa	43.61	1	43.61	47.72	4.11
27	aicrp 2018 14.spa	38.91	0	38.91	43.59	4.68
28	aicrp 2018 15.spa	37.70	0	37.70	37.92	0.22
29	aicrp 2018 16.spa	40.38	0	40.38	46.32	5.94
30	aicrp 2018 17.spa	48.65	0	48.65	55.01	6.36
31	aicrp 2018 18.spa	45.19	1	45.19	39.57	-5.62
32	aicrp 2018 19.spa	11.60	0	11.60	14.90	3.30
33	aicrp 2018 20.spa	48.40	0	48.40	53.41	5.01
34	aicrp 2018 21.spa	41.42	0	41.42	40.12	-1.30
35	aicrp 2018 22.spa	42.58	0	42.58	53.57	10.99
37	aicrp 2023 1 r .sp	40.61	0	40.61	37.16	-3.45
40	aicrp 2023 19 r .s	41.66	0	41.66	46.88	5.22
42	aicrp 2023 6 r sa	45.51	0	45.51	45.57	0.06
44	aicrp 2023 24 r .s	51.93	0	51.93	56.32	4.39
46	aicrp 2023 18 r .s	47.83	1	47.83	57.00	9.17
47	aicrp 2023 26 r .s	12.61	0	12.61	16.25	3.64
48	aicrp 2023 12 r .	44.88	0	44.88	49.19	4.31
50	aicrp 2023 21 r .s	44.72	1	44.72	50.64	5.92
53	aicrp 2023 10 r .	44.50	0	44.50	48.90	4.40
54	aicrp 2023 8 r .s	40.85	0	40.85	40.45	-0.40
57	aicrp 2023 4 r .s	40.40	0	40.40	37.33	-3.07
58	aicrp 2023 3 r .s	41.74	0	41.74	38.97	-2.77
60	aicrp 2021 2.spa	41.06	0	41.06	36.57	-4.49
61	aicrp 2021 3.spa	38.07	0	38.07	45.13	7.06
63	aicrp 2021 5.spa	40.85	0	40.85	39.45	-1.40
64	aicrp 2021 6.spa	42.07	0	42.07	36.47	-5.60
65	aicrp 2021 7.spa	12.26	0	12.26	13.21	0.95
66	aicrp 2021 8.spa	37.73	0	37.73	38.20	0.47
68	aicrp 2021 10.spa	45.87	0	45.87	40.57	-5.30
70	aicrp 2021 12.spa	40.09	0	40.09	41.40	1.31
72	aicrp 2021 14.spa	43.92	0	43.92	42.77	-1.15

73	aicrp 2021 15.spa	43.93	0	43.93	36.78	-7.15
74	aicrp 2021 16.spa	40.79	1	40.79	45.55	4.76
75	aicrp 2021 17.spa	52.81	0	52.81	47.26	-5.55
76	aicrp 2023 6	45.22	0	45.22	42.21	-3.01
78	aicrp 2023 9	47.25	0	47.25	43.68	-3.57
79	aicrp 2023 11.spa	46.85	0	46.85	45.65	-1.20
82	aicrp 2023 12.spa	48.05	0	48.05	45.05	-3.00
83	aicrp 2023 3.spa	44.77	0	44.77	46.79	2.02
84	aicrp 2023 14.spa	46.53	0	46.53	45.73	-0.80
85	aicrp 2023 2	49.17	0	49.17	39.75	-9.42
86	aicrp 2023 13.spa	48.28	0	48.28	45.26	-3.02
87	aicrp 2023 5.spa	46.11	0	46.11	46.81	0.70
88	aicrp 2023 1.spa	47.12	0	47.12	38.42	-8.70
89	aicrp 2023 18	49.17	0	49.17	45.17	-4.00
94	aicrp 2023 20.spa	49.16	0	49.16	42.35	-6.81
1	2017 1.spa	11.75	3	11.75	22.49	10.74
3	2017 3.spa	47.90	3	47.90	28.90	-19.00
6	2017 6.spa	9.75	3	9.75	31.98	22.23
16	aicrp 2018 3.spa	40.18	3	40.18	51.46	11.28
36	aicrp 2023 17 r s	32.60	3	32.60	53.09	20.49
38	aicrp 2023 25 r .sp	10.94	3	10.94	44.84	33.90
39	aicrp 2023 22 r	43.05	3	43.05	17.07	-25.98
41	aicrp 2023 2 r .s	42.40	3	42.40	29.77	-12.63
43	aicrp 2023 14 r	11.47	3	11.47	44.11	32.64
45	aicrp 2023 11 r.sp	13.58	3	13.58	32.27	18.69
49	aicrp 2023 23 r .	44.90	3	44.90	22.76	-22.14
51	aicrp 2023 20 r .	44.61	3	44.61	23.49	-21.12
52	aicrp 2023 16 r .	35.62	3	35.62	52.16	16.54
55	aicrp 2023 7 r .s	11.55	3	11.55	47.57	36.02
56	aicrp 2023 5 r .s	45.02	3	45.02	27.63	-17.39
59	aicrp 2021 1 samp	46.15	3	46.15	33.32	-12.83
62	aicrp 2021 4.spa	47.01	3	47.01	36.33	-10.68
67	aicrp 2021 9.spa	39.32	3	39.32	26.26	-13.06

69	aicrp 2021	11.spa	44.87	3	44.87	30.90	-13.97
71	aicrp 2021	13.spa	47.66	3	47.66	36.40	-11.26
77	aicrp 2023	8.spa	44.14	3	44.14	10.33	-33.81
80	aicrp 2023	10	13.53	3	13.53	33.91	20.38
81	aicrp 2023	4.spa	46.49	3	46.49	36.18	-10.31
90	aicrp 2023	17.spa	44.55	3	44.55	24.48	-20.07
91	aicrp 2023	19.spa	54.24	3	54.24	43.94	-10.30
92	aicrp 2023	7.spa	46.26	3	46.26	35.93	-10.33
93	aicrp 2023	16.spa	11.46	3	11.46	40.75	29.29
