# Supplementary material for: Use of near-infrared spectroscopy for screening the oil content, protein, phytic acid, glucosinolates, and fatty acid profile in oilseed Brassica species
Source: Front Nutr. 2025 Sep 2;12:1632421. doi: 10.3389/fnut.2025.1632421 (PMC12439716; doi:10.3389/fnut.2025.1632421)
Supplement: Supplementary file 8 [file Data_Sheet_8.pdf]

C:\RESULT Data\Workflows\Fatty acid table 28 (R3) Palmitic.qnt
Revision: 1 Last saved on: Wed Aug 28 10:15:14 2024
Printed on: Thu Feb 06 16:40:48 2025

Calculated vs Actual Plot - palmitic

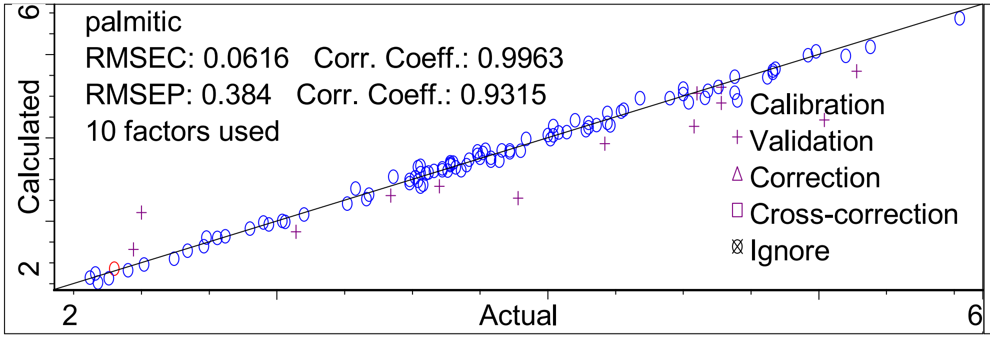

Difference vs Actual Plot - palmitic

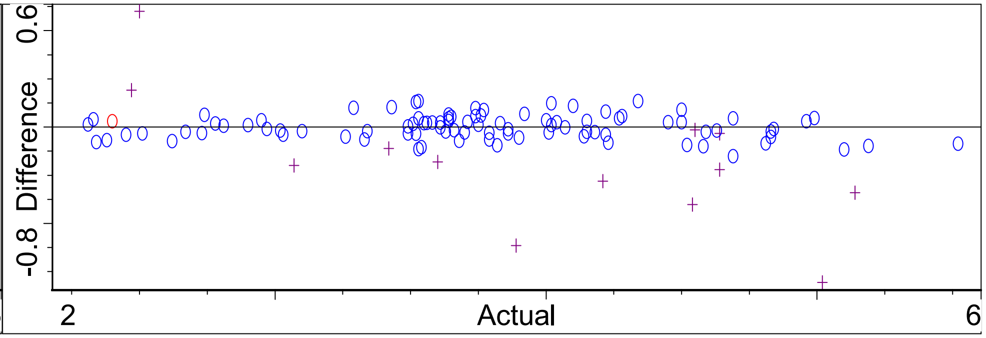

10 factors used

Calibration Results Table - palmitic

Index	File Name	Spectrum Title	Usage	Actual	Calculated	Diff. x Path
1	2017 1.spa	2017 1.spa	0	2.40	2.43	0.03
2	2017 2.spa	2017 2.spa	0	2.95	2.98	0.03
3	2017 3.spa	2017 3.spa	0	4.50	4.59	0.09
4	2017 4.spa	2017 4.spa	0	4.52	4.43	-0.09
5	2017 5.spa	2017 5.spa	0	4.18	4.15	-0.03
6	2017 6.spa	2017 6.spa	0	2.67	2.64	-0.03
7	2017 7.spa	2017 7.spa	0	4.45	4.47	0.02
8	2017 8.spa	2017 8.spa	0	4.28	4.34	0.06
9	2017 9.spa	2017 9.spa	0	4.99	5.04	0.05
10	2017 10.spa	2017 10.spa	0	3.56	3.58	0.02
11	2017 13.spa	2017 13.spa	0	2.90	2.91	0.01
12	2017 14.spa	2017 14.spa	0	4.59	4.56	-0.03
13	2017 15.spa	2017 15.spa	0	2.73	2.70	-0.03
14	aicrp 2018 1.spa	aicrp 2018 1.spa	0	3.79	3.73	-0.06
15	aicrp 2018 2.spa	aicrp 2018 2.spa	0	3.55	3.57	0.02
16	aicrp 2018 3.spa	aicrp 2018 3.spa	0	3.74	3.84	0.10
17	aicrp 2018 4.spa	aicrp 2018 4.spa	0	3.52	3.48	-0.04
18	aicrp 2018 5.spa	aicrp 2018 5.spa	0	4.81	4.72	-0.09
19	aicrp 2018 6.spa	aicrp 2018 6.spa	0	4.22	4.18	-0.04

20	aicrp 2018 7.spa	aicrp 2018 7.spa	0	4.27	4.32	0.05
21	aicrp 2018 8.spa	aicrp 2018 8.spa	0	3.63	3.61	-0.02
23	aicrp 2018 10 .spa	aicrp 2018 10 .spa	0	3.53	3.57	0.04
24	aicrp 2018 11.spa	aicrp 2018 11.spa	0	3.64	3.71	0.07
25	aicrp 2018 12.spa	aicrp 2018 12.spa	0	3.77	3.86	0.09
26	aicrp 2018 13.spa	aicrp 2018 13.spa	0	3.92	3.99	0.07
27	aicrp 2018 14.spa	aicrp 2018 14.spa	0	4.84	4.83	-0.01
28	aicrp 2018 15.spa	aicrp 2018 15.spa	0	3.43	3.53	0.10
29	aicrp 2018 16.spa	aicrp 2018 16.spa	0	4.58	4.48	-0.10
30	aicrp 2018 17.spa	aicrp 2018 17.spa	0	4.23	4.15	-0.08
31	aicrp 2018 18.spa	aicrp 2018 18.spa	0	4.07	4.07	-0.00
32	aicrp 2018 19.spa	aicrp 2018 19.spa	1	2.47	2.66	0.19
33	aicrp 2018 20.spa	aicrp 2018 20.spa	0	3.83	3.85	0.02
34	aicrp 2018 21.spa	aicrp 2018 21.spa	1	4.64	4.61	-0.03
35	aicrp 2018 22.spa	aicrp 2018 22.spa	0	3.71	3.74	0.03
36	aicrp 2023 17 r s	aicrp 2023 17 r sample.spa	0	3.82	3.73	-0.09
37	aicrp 2023 1 r .s	aicrp 2023 1 r .spa	0	5.19	5.09	-0.10
39	aicrp 2023 22 r	aicrp 2023 22 r sample.spa	0	4.01	3.98	-0.03
40	aicrp 2023 19 r .	aicrp 2023 19 r .spa	0	4.83	4.78	-0.05
41	aicrp 2023 2 r .s	aicrp 2023 2 r .spa	0	4.96	4.99	0.03
42	aicrp 2023 6 r s	aicrp 2023 6 r sample.spa	0	4.63	4.61	-0.02
44	aicrp 2023 24 r .s	aicrp 2023 24 r .spa	0	4.50	4.53	0.03
45	aicrp 2023 11 r.sp	aicrp 2023 11 r.spa	0	2.33	2.37	0.04
46	aicrp 2023 18 r .	aicrp 2023 18 r .spa	1	5.02	4.21	-0.81
47	aicrp 2023 26 r .	aicrp 2023 26 r .spa	0	2.34	2.26	-0.08
48	aicrp 2023 12 r	aicrp 2023 12 r .spa	0	4.15	4.18	0.03
49	aicrp 2023 23 r .	aicrp 2023 23 r .spa	0	3.68	3.61	-0.07
50	aicrp 2023 21 r .s	aicrp 2023 21 r .spa	0	4.22	4.30	0.08
51	aicrp 2023 20 r	aicrp 2023 20 r .spa	1	3.89	3.28	-0.61
53	aicrp 2023 10 r	aicrp 2023 10 r .spa	0	3.75	3.76	0.01
54	aicrp 2023 8 r .s	aicrp 2023 8 r .spa	1	4.64	4.42	-0.22
55	aicrp 2023 7 r .s	aicrp 2023 7 r .spa	0	2.38	2.31	-0.07
56	aicrp 2023 5 r .s	aicrp 2023 5 r .spa	0	3.51	3.53	0.02

57	aicrp 2023 4 r .saicrp 2023 4 r .spa	0	4.69	4.54	-0.15
58	aicrp 2023 3 r .saicrp 2023 3 r .spa	0	5.52	5.43	-0.09
59	aicrp 2021 1 sampaicrp 2021 1 sample.spa	0	4.34	4.47	0.13
60	aicrp 2021 2.spa aicrp 2021 2.spa	0	4.83	4.81	-0.02
61	aicrp 2021 3.spa aicrp 2021 3.spa	1	5.14	4.80	-0.34
62	aicrp 2021 4.spa aicrp 2021 4.spa	1	4.54	4.14	-0.40
63	aicrp 2021 5.spa aicrp 2021 5.spa	0	3.90	3.84	-0.06
64	aicrp 2021 6.spa aicrp 2021 6.spa	0	3.76	3.82	0.06
65	aicrp 2021 7.spa aicrp 2021 7.spa	0	3.02	3.00	-0.02
66	aicrp 2021 8.spa aicrp 2021 8.spa	0	3.10	3.08	-0.02
67	aicrp 2021 9.spa aicrp 2021 9.spa	1	3.60	3.42	-0.18
68	aicrp 2021 10.spa aicrp 2021 10.spa	0	3.49	3.49	0.00
69	aicrp 2021 11.spa aicrp 2021 11.spa	0	3.70	3.67	-0.03
70	aicrp 2021 12.spa aicrp 2021 12.spa	0	5.10	4.98	-0.12
71	aicrp 2021 13.spa aicrp 2021 13.spa	0	4.00	4.03	0.03
72	aicrp 2021 14.spa aicrp 2021 14.spa	0	3.34	3.32	-0.02
73	aicrp 2021 15.spa aicrp 2021 15.spa	0	4.69	4.73	0.04
74	aicrp 2021 16.spa aicrp 2021 16.spa	1	4.55	4.54	-0.01
75	aicrp 2021 17.spa aicrp 2021 17.spa	0	3.66	3.64	-0.02
76	aicrp 2023 6 aicrp 2023 6 .spa	0	3.53	3.42	-0.11
77	aicrp 2023 8.spa aicrp 2023 8.spa	0	3.52	3.65	0.13
78	aicrp 2023 9 aicrp 2023 9 .spa	0	3.74	3.80	0.06
79	aicrp 2023 11.spaaicrp 2023 11.spa	0	4.15	4.13	-0.02
81	aicrp 2023 4.spa aicrp 2023 4.spa	0	4.14	4.09	-0.05
82	aicrp 2023 12.spa aicrp 2023 12.spa	0	3.58	3.60	0.02
83	aicrp 2023 3.spa aicrp 2023 3.spa	0	4.02	4.14	0.12
84	aicrp 2023 14.spaaicrp 2023 14.spa	0	3.29	3.39	0.10
85	aicrp 2023 2 aicrp 2023 2 .spa	0	3.61	3.63	0.02
86	aicrp 2023 13.spaaicrp 2023 13.spa	0	2.97	2.96	-0.01
87	aicrp 2023 5.spa aicrp 2023 5.spa	0	3.49	3.46	-0.03
88	aicrp 2023 1.spa aicrp 2023 1.spa	0	3.64	3.69	0.05
89	aicrp 2023 18 aicrp 2023 18 .spa	0	3.86	3.85	-0.01
90	aicrp 2023 17.spa aicrp 2023 17.spa	0	3.65	3.70	0.05

91	aicrp 2023 19.spa	aicrp 2023 19.spa	0	3.61	3.61	-0.00
92	aicrp 2023 7.spa	aicrp 2023 7.spa	0	3.53	3.67	0.14
94	aicrp 2023 20.spa	aicrp 2023 20.spa	0	3.86	3.83	-0.03
95	pm-21.spa	Sample 2024-08-05 145342 GMT+0530	1	4.21	3.93	-0.28
96	ij-31 sd.spa	Sample 2024-08-05 143912 GMT+0530	0	2.74	2.80	0.06
97	nrcbh 101.spa	Sample 2024-08-05 143728 GMT+0530	0	3.33	3.27	-0.06
98	pm-30 sd.spa	Sample 2024-08-05 144217 GMT+0530	1	3.07	2.87	-0.20
99	pdz-1 r1.spa	Sample 2024-08-05 144657 GMT+0530	0	3.54	3.44	-0.10
100	rgn-73 .spa	Sample 2024-08-05 145009 GMT+0530	1	3.42	3.31	-0.11
101	pm-29.spa	Sample 2024-08-05 145201 GMT+0530	0	4.10	4.21	0.11
102	pm-21 r.spa	Sample 2024-08-05 145646 GMT+0530	0	2.45	2.41	-0.04
103	pm-29 r.spa	Sample 2024-08-05 145802 GMT+0530	0	2.78	2.80	0.02
104	rgn-73 r.spa	Sample 2024-08-05 145935 GMT+0530	0	4.02	4.03	0.01
105	pdz-1 r.spa	Sample 2024-08-05 150053 GMT+0530	0	3.64	3.67	0.03
106	pm-30 r .spa	Sample 2024-08-05 150211 GMT+0530	0	3.79	3.76	-0.03
107	ij-31 r.spa	Sample 2024-08-05 150352 GMT+0530	0	2.31	2.32	0.01
108	nrcbh-101 r.spa	Sample 2024-08-05 150501 GMT+0530	0	2.51	2.48	-0.03
109	nrcbh- 101 r1.spa	Sample 2024-08-05 150710 GMT+0530	1	2.50	3.10	0.60
110	ij-31 r1.spa	Sample 2024-08-05 150832 GMT+0530	0	2.81	2.82	0.01
111	pm-30 r1.spa	Sample 2024-08-05 150941 GMT+0530	0	3.03	2.99	-0.04
112	pdz-1 r1.spa	Sample 2024-08-05 151055 GMT+0530	0	3.26	3.21	-0.05
113	pm-29 r1.spa	Sample 2024-08-05 151343 GMT+0530	0	4.04	4.06	0.02
114	pm-21 r1.spa	Sample 2024-08-05 151454 GMT+0530	0	2.62	2.55	-0.07
22	aicrp 2018 9.spa	aicrp 2018 9.spa	3	1.90	3.44	1.54
38	aicrp 2023 25 r .spa	aicrp 2023 25 r .spa	3	2.02	3.80	1.78
43	aicrp 2023 14 r	aicrp 2023 14 r sample.spa	3	2.04	3.24	1.20
52	aicrp 2023 16 r .	aicrp 2023 16 r .spa	3	5.45	4.41	-1.04
80	aicrp 2023 10	aicrp 2023 10 .spa	3	2.19	3.46	1.27
93	aicrp 2023 16.spa	aicrp 2023 16.spa	3	1.95	3.18	1.23
