# Supplementary material for: Use of near-infrared spectroscopy for screening the oil content, protein, phytic acid, glucosinolates, and fatty acid profile in oilseed Brassica species
Source: Front Nutr. 2025 Sep 2;12:1632421. doi: 10.3389/fnut.2025.1632421 (PMC12439716; doi:10.3389/fnut.2025.1632421)
Supplement: Supplementary file 9 [file Data_Sheet_9.pdf]

MUSTARD SEED PHYTIC ACID   C:\My Documents\Omnic\quant\PHYTIC ACID 6-2-25.qnt
Revision: 1   Last saved on: Thu Feb 06 16:11:06 2025
Printed on: Thu Feb 06 16:21:56 2025

Calculated vs Actual Plot - PHYTIC ACID

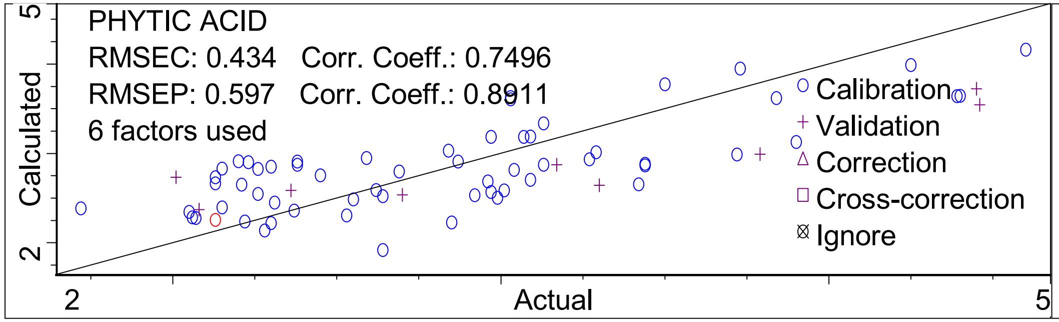

Difference vs Actual Plot - PHYTIC ACID

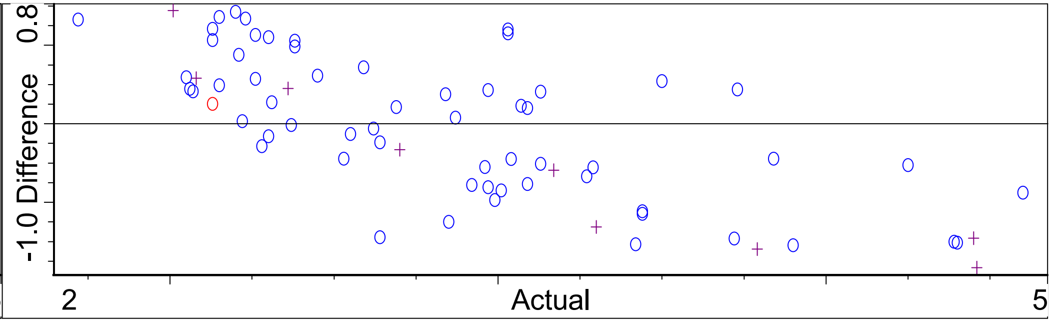

6 factors used

Calibration Results Table - PHYTIC ACID

Index	File Name	Spectrum Title	Usage	Actual	Calculated	Diff. x Path
1	2017 1.spa	Sample 2024-07-01 101644 GMT+0530	0	2.13	2.26	0.13
2	2017 2.spa	Sample 2024-07-01 101731 GMT+0530	1	2.36	2.59	0.23
3	2017 3.spa	Sample 2024-07-01 101818 GMT+0530	0	2.26	2.83	0.57
4	2017 4.spa	Sample 2024-07-01 101909 GMT+0530	0	2.05	2.35	0.30
5	2017 5.spa	Sample 2024-07-01 102150 GMT+0530	0	2.53	2.31	-0.22
6	2017 6.spa	Sample 2024-07-01 102238 GMT+0530	0	2.23	2.90	0.67
7	2017 7.spa	Sample 2024-07-01 102324 GMT+0530	0	2.38	2.91	0.53
8	2017 8.spa	Sample 2024-07-01 102410 GMT+0530	0	2.07	2.28	0.21
9	2017 9.spa	Sample 2024-07-01 102457 GMT+0530	0	2.31	2.45	0.14
10	2017 10.spa	Sample 2024-07-01 102555 GMT+0530	0	1.72	2.38	0.66
11	2017 13.spa	Sample 2024-07-01 102640 GMT+0530	0	2.26	2.55	0.29
12	2017 14.spa	Sample 2024-07-01 102730 GMT+0530	0	2.22	2.24	0.02
13	2017 15.spa	Sample 2024-07-01 102812 GMT+0530	0	2.30	2.22	-0.08
14	aicrp 2023 6	Sample 2024-07-01 103633 GMT+0530	0	3.73	3.95	0.22
15	aicrp 2023 8.spa	Sample 2024-07-01 103829 GMT+0530	1	4.46	3.54	-0.92
16	aicrp 2023 9	Sample 2024-07-01 103923 GMT+0530	0	4.39	3.64	-0.75
17	aicrp 2023 11.spa	Sample 2024-07-01 104008 GMT+0530	0	3.07	3.18	0.11
18	aicrp 2023 10	Sample 2024-07-01 104201 GMT+0530	0	3.03	3.63	0.60

MUSTARD SEED PHYTIC ACID C:\My Documents\Omnic\quant\PHYTIC ACID 6-2-25.qnt
Revision: 1 Last saved on: Thu Feb 06 16:11:06 2025
Printed on: Thu Feb 06 16:21:56 2025

19	aicrp 2023	4.spa	Sample 2024-07-01 104338 GMT+0530	0	4.60	4.16	-0.44
20	aicrp 2023	12.spa	Sample 2024-07-01 104434 GMT+0530	0	3.13	3.34	0.21
21	aicrp 2023	3.spa	Sample 2024-07-01 104541 GMT+0530	1	3.17	2.87	-0.30
22	aicrp 2023	14.spa	Sample 2024-07-01 104627 GMT+0530	0	3.84	3.62	-0.22
23	aicrp 2023	2	Sample 2024-07-01 104720 GMT+0530	0	4.40	3.64	-0.76
24	aicrp 2023	13.spa	Sample 2024-07-01 104821 GMT+0530	1	4.45	3.72	-0.73
25	aicrp 2023	5.spa	Sample 2024-07-01 104912 GMT+0530	0	4.25	3.99	-0.26
28	aicrp 2023	17.spa	Sample 2024-07-01 105458 GMT+0530	0	3.90	3.13	-0.77
30	aicrp 2023	7.spa	Sample 2024-07-01 105658 GMT+0530	0	3.50	3.77	0.27
31	aicrp 2023	16.spa	Sample 2024-07-01 105755 GMT+0530	0	3.72	2.99	-0.73
32	aicrp 2023	20.spa	Sample 2024-07-01 105857 GMT+0530	0	3.03	3.61	0.58
33	aicrp 2023	9 r .s	Sample 2024-07-01 110402 GMT+0530	0	3.44	2.87	-0.57
34	aicrp 2023	17 r .s	Sample 2024-07-01 110546 GMT+0530	0	3.29	3.01	-0.28
35	aicrp 2023	17 r s	Sample 2024-07-01 110639 GMT+0530	1	3.30	2.64	-0.66
36	aicrp 2023	1 r .s	Sample 2024-07-01 110747 GMT+0530	1	2.01	2.73	0.72
37	aicrp 2023	25 r .sp	Sample 2024-07-01 110839 GMT+0530	0	2.37	2.36	-0.01
38	aicrp 2023	22 r	Sample 2024-07-01 110952 GMT+0530	1	2.08	2.37	0.29
39	aicrp 2023	19 r .	Sample 2024-07-01 111050 GMT+0530	0	2.28	2.14	-0.14
40	aicrp 2023	2 r .s	Sample 2024-07-01 111201 GMT+0530	1	2.70	2.54	-0.16
41	aicrp 2018	10 .spa	Sample 2024-06-28 151205 GMT+0530	0	3.27	2.94	-0.33
42	aicrp 2018	11.spa	Sample 2024-06-28 151404 GMT+0530	0	3.09	2.71	-0.38
43	aicrp 2018	12.spa	Sample 2024-06-28 151502 GMT+0530	0	2.84	3.03	0.19
44	aicrp 2018	13.spa	Sample 2024-06-28 151557 GMT+0530	0	3.42	2.65	-0.77
46	aicrp 2018	15.spa	Sample 2024-06-28 151847 GMT+0530	0	2.96	2.69	-0.27
47	aicrp 2018	16.spa	Sample 2024-06-28 152020 GMT+0530	0	2.15	2.39	0.24
48	aicrp 2018	17.spa	Sample 2024-06-28 152204 GMT+0530	0	2.59	2.95	0.36
49	aicrp 2018	18.spa	Sample 2024-06-28 152259 GMT+0530	0	3.13	2.87	-0.26
50	aicrp 2018	19.spa	Sample 2024-06-28 152439 GMT+0530	0	3.09	3.19	0.10
51	aicrp 2018	20.spa	Sample 2024-06-28 152639 GMT+0530	0	2.64	1.92	-0.72
52	aicrp 2018	20 .spa	Sample 2024-06-28 152742 GMT+0530	0	2.64	2.52	-0.12
53	aicrp 2018	21.spa	Sample 2024-06-28 152928 GMT+0530	0	2.30	2.85	0.55
54	aicrp 2018	22.spa	Sample 2024-06-28 153019 GMT+0530	0	2.20	2.91	0.71

MUSTARD SEED PHYTIC ACID C:\My Documents\Omnic\quant\PHYTIC ACID 6-2-25.qnt
Revision: 1 Last saved on: Thu Feb 06 16:11:06 2025
Printed on: Thu Feb 06 16:21:56 2025

55	aicrp 2021 1 samp	Sample 2024-06-28 102909 GMT+0530	0	2.55	2.49	-0.06
56	aicrp 2021 2.spa	Sample 2024-06-28 103249 GMT+0530	0	2.85	2.23	-0.62
57	aicrp 2021 3.spa	Sample 2024-06-28 103439 GMT+0530	0	3.04	2.82	-0.22
58	aicrp 2021 4.spa	Sample 2024-06-28 103558 GMT+0530	0	2.92	2.53	-0.39
59	aicrp 2021 5.spa	Sample 2024-06-28 103658 GMT+0530	0	2.38	2.87	0.49
60	aicrp 2021 6.spa	Sample 2024-06-28 103810 GMT+0530	0	3.01	2.59	-0.42
61	aicrp 2021 7.spa	Sample 2024-06-28 103922 GMT+0530	0	2.87	2.91	0.04
63	aicrp 2021 9.spa	Sample 2024-06-28 104111 GMT+0530	0	3.44	2.88	-0.56
65	aicrp 2021 11.spa	Sample 2024-06-28 104332 GMT+0530	0	2.15	2.83	0.68
66	aicrp 2021 12.spa	Sample 2024-06-28 104424 GMT+0530	0	2.99	2.50	-0.49
67	aicrp 2021 13.spa	Sample 2024-06-28 104526 GMT+0530	1	3.79	2.99	-0.80
68	aicrp 2021 14.spa	Sample 2024-06-28 104626 GMT+0530	0	2.13	2.66	0.53
69	aicrp 2021 15.spa	Sample 2024-06-28 104819 GMT+0530	0	2.13	2.73	0.60
70	aicrp 2021 16.spa	Sample 2024-06-28 104920 GMT+0530	0	2.06	2.28	0.22
75	aicrp 2018 4.spa	Sample 2024-06-28 110154 GMT+0530	0	2.69	2.80	0.11
76	aicrp 2018 5.spa	Sample 2024-06-28 110243 GMT+0530	0	2.21	2.65	0.44
77	aicrp 2018 6.spa	Sample 2024-06-28 110352 GMT+0530	0	2.62	2.59	-0.03
78	aicrp 2018 7.spa	Sample 2024-06-28 110435 GMT+0530	0	2.97	2.57	-0.40
79	aicrp 2018 8.spa	Sample 2024-06-28 110521 GMT+0530	0	2.97	3.19	0.22
80	aicrp 2018 9.spa	Sample 2024-06-28 110943 GMT+0530	0	2.45	2.76	0.31
26	aicrp 2023 1.spa	Sample 2024-07-01 105049 GMT+0530	3	4.92	3.79	-1.13
27	aicrp 2023 18	Sample 2024-07-01 105209 GMT+0530	3	4.03	2.94	-1.09
29	aicrp 2023 19.spa	Sample 2024-07-01 105554 GMT+0530	3	5.00	3.45	-1.55
45	aicrp 2018 14.spa	Sample 2024-06-28 151802 GMT+0530	3	3.43	2.47	-0.96
62	aicrp 2021 8.spa	Sample 2024-06-28 104016 GMT+0530	3	4.00	3.00	-1.00
64	aicrp 2021 10.spa	Sample 2024-06-28 104212 GMT+0530	3	2.00	3.10	1.10
71	aicrp 2021 17.spa	Sample 2024-06-28 105029 GMT+0530	3	2.02	3.04	1.02
72	aicrp 2018 1.spa	Sample 2024-06-28 105808 GMT+0530	3	2.15	3.33	1.18
73	aicrp 2018 2.spa	Sample 2024-06-28 105951 GMT+0530	3	1.86	2.98	1.12
74	aicrp 2018 3.spa	Sample 2024-06-28 110045 GMT+0530	3	1.97	2.93	0.96
