# Supplementary material for: Use of near-infrared spectroscopy for screening the oil content, protein, phytic acid, glucosinolates, and fatty acid profile in oilseed Brassica species
Source: Front Nutr. 2025 Sep 2;12:1632421. doi: 10.3389/fnut.2025.1632421 (PMC12439716; doi:10.3389/fnut.2025.1632421)
Supplement: Supplementary file 10 [file Data_Sheet_10.pdf]

mustard seed protein   C:\My Documents\Omnic\quant\protein 6-2-25.qnt
Revision: 2   Last saved on: Thu Feb 06 16:51:13 2025
Printed on: Tue Feb 11 11:13:30 2025

Calculated vs Actual Plot - protein

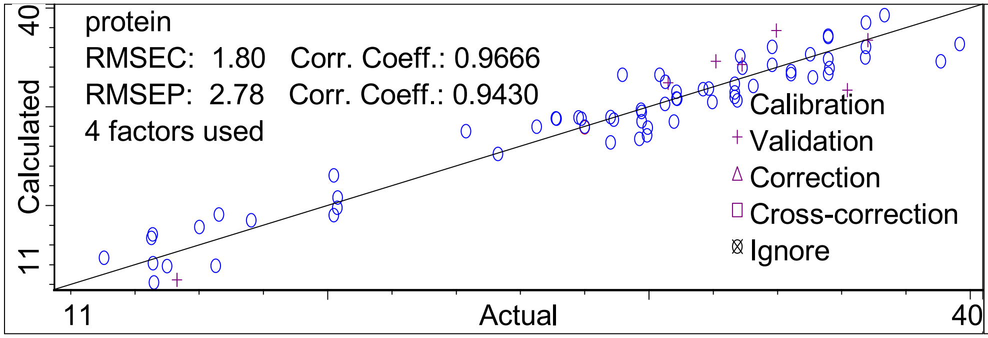

Difference vs Actual Plot - protein

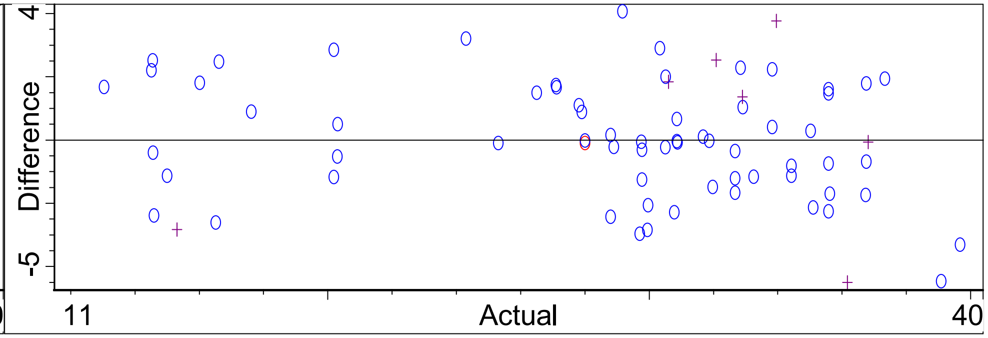

4 factors used

Calibration Results Table - protein

Index	Spectrum Title	Usage	Actual	Calculated	Diff. x Path
1	Sample 2024-07-01 101644 GMT+0530	0	28.00	27.91	-0.09
2	Sample 2024-07-01 101731 GMT+0530	0	28.80	26.38	-2.42
3	Sample 2024-07-01 101818 GMT+0530	0	29.70	26.74	-2.96
4	Sample 2024-07-01 101909 GMT+0530	0	29.75	29.69	-0.06
5	Sample 2024-07-01 102150 GMT+0530	1	30.60	32.44	1.84
6	Sample 2024-07-01 102238 GMT+0530	0	26.50	27.99	1.49
7	Sample 2024-07-01 102324 GMT+0530	0	28.80	28.96	0.16
8	Sample 2024-07-01 102410 GMT+0530	0	27.12	28.79	1.67
9	Sample 2024-07-01 102457 GMT+0530	0	27.10	28.83	1.73
11	Sample 2024-07-01 102640 GMT+0530	0	24.30	27.51	3.21
13	Sample 2024-07-01 102812 GMT+0530	0	25.30	25.21	-0.09
14	Sample 2024-07-01 103633 GMT+0530	0	28.00	27.99	-0.01
15	Sample 2024-07-01 103829 GMT+0530	0	27.81	28.91	1.10
16	Sample 2024-07-01 103923 GMT+0530	0	33.83	34.25	0.42
17	Sample 2024-07-01 104008 GMT+0530	0	34.42	33.61	-0.81
18	Sample 2024-07-01 104201 GMT+0530	0	36.73	34.99	-1.74
19	Sample 2024-07-01 104338 GMT+0530	0	35.10	32.97	-2.13
20	Sample 2024-07-01 104434 GMT+0530	0	37.33	39.27	1.94
21	Sample 2024-07-01 104541 GMT+0530	1	36.17	31.66	-4.51

22	Sample 2024-07-01 104627 GMT+0530 0	35.58	33.33	-2.25
23	Sample 2024-07-01 104720 GMT+0530 0	33.25	32.09	-1.16
24	Sample 2024-07-01 104821 GMT+0530 0	39.67	36.36	-3.31
25	Sample 2024-07-01 104912 GMT+0530 0	35.02	35.31	0.29
26	Sample 2024-07-01 105049 GMT+0530 0	36.75	38.54	1.79
27	Sample 2024-07-01 105209 GMT+0530 0	35.58	34.83	-0.75
28	Sample 2024-07-01 105458 GMT+0530 0	36.75	36.07	-0.68
29	Sample 2024-07-01 105554 GMT+0530 0	35.58	37.05	1.47
30	Sample 2024-07-01 105658 GMT+0530 0	34.42	33.29	-1.13
31	Sample 2024-07-01 105755 GMT+0530 0	32.67	31.00	-1.67
32	Sample 2024-07-01 105857 GMT+0530 0	33.83	36.07	2.24
33	Sample 2024-07-01 110402 GMT+0530 0	39.08	34.62	-4.46
34	Sample 2024-07-01 110546 GMT+0530 1	32.08	34.61	2.53
35	Sample 2024-07-01 110639 GMT+0530 0	35.58	37.19	1.61
36	Sample 2024-07-01 110747 GMT+0530 0	29.17	33.24	4.07
37	Sample 2024-07-01 110839 GMT+0530 0	30.33	33.24	2.91
38	Sample 2024-07-01 110952 GMT+0530 0	32.67	31.46	-1.21
39	Sample 2024-07-01 111050 GMT+0530 1	36.81	36.75	-0.06
40	Sample 2024-07-01 111201 GMT+0530 0	20.19	23.04	2.85
43	Sample 2024-07-01 111438 GMT+0530 0	29.96	27.89	-2.07
45	Sample 2024-07-01 111616 GMT+0530 0	29.77	29.46	-0.31
47	Sample 2024-07-01 111809 GMT+0530 0	29.77	28.52	-1.25
48	Sample 2024-07-01 111901 GMT+0530 0	27.90	28.78	0.88
51	Sample 2024-07-01 112521 GMT+0530 0	31.68	31.79	0.11
53	Sample 2024-07-01 112725 GMT+0530 0	31.86	31.83	-0.03
54	Sample 2024-07-01 112847 GMT+0530 1	33.96	37.73	3.77
55	Sample 2024-07-01 113001 GMT+0530 0	32.91	33.95	1.04
56	Sample 2024-07-01 113054 GMT+0530 0	32.67	32.32	-0.35
57	Sample 2024-07-01 113146 GMT+0530 0	32.84	35.12	2.28
58	Sample 2024-06-28 151205 GMT+0530 1	32.90	34.27	1.37
59	Sample 2024-06-28 151404 GMT+0530 0	30.86	31.52	0.66
60	Sample 2024-06-28 151502 GMT+0530 0	31.97	30.49	-1.48
61	Sample 2024-06-28 151557 GMT+0530 0	30.88	30.81	-0.07

62	Sample 2024-06-28 151802 GMT+0530 0	29.94	27.10	-2.84
63	Sample 2024-06-28 151847 GMT+0530 0	30.86	30.83	-0.03
65	Sample 2024-06-28 152204 GMT+0530 0	30.78	28.49	-2.29
66	Sample 2024-06-28 152259 GMT+0530 0	14.55	17.07	2.52
67	Sample 2024-06-28 152439 GMT+0530 0	14.56	14.16	-0.40
68	Sample 2024-06-28 152639 GMT+0530 0	16.01	17.82	1.81
69	Sample 2024-06-28 152742 GMT+0530 1	15.31	12.47	-2.84
72	Sample 2024-06-28 102909 GMT+0530 0	16.51	13.90	-2.61
76	Sample 2024-06-28 103658 GMT+0530 0	16.61	19.09	2.48
77	Sample 2024-06-28 103810 GMT+0530 0	13.04	14.72	1.68
78	Sample 2024-06-28 103922 GMT+0530 0	20.19	19.02	-1.17
79	Sample 2024-06-28 104016 GMT+0530 0	17.62	18.51	0.89
80	Sample 2024-06-28 104111 GMT+0530 0	15.00	13.87	-1.13
82	Sample 2024-06-28 104332 GMT+0530 0	14.59	12.20	-2.39
83	Sample 2024-06-28 104424 GMT+0530 0	14.51	16.72	2.21
89	Sample 2024-06-28 105808 GMT+0530 0	20.31	20.81	0.50
90	Sample 2024-06-28 105951 GMT+0530 0	30.51	32.51	2.00
91	Sample 2024-06-28 110045 GMT+0530 0	35.62	33.92	-1.70
94	Sample 2024-06-28 110352 GMT+0530 0	28.90	28.68	-0.22
95	Sample 2024-06-28 110435 GMT+0530 0	30.50	30.27	-0.23
97	Sample 2024-06-28 110943 GMT+0530 0	20.30	19.78	-0.52
10	Sample 2024-07-01 102555 GMT+0530 3	26.25	37.74	11.49
12	Sample 2024-07-01 102730 GMT+0530 3	23.60	27.30	3.70
41	Sample 2024-07-01 111251 GMT+0530 3	14.95	33.07	18.12
42	Sample 2024-07-01 111339 GMT+0530 3	13.49	31.52	18.03
44	Sample 2024-07-01 111523 GMT+0530 3	14.15	21.59	7.44
46	Sample 2024-07-01 111708 GMT+0530 3	14.51	31.78	17.27
49	Sample 2024-07-01 111955 GMT+0530 3	13.95	32.11	18.16
50	Sample 2024-07-01 112144 GMT+0530 3	14.60	27.69	13.09
52	Sample 2024-07-01 112629 GMT+0530 3	30.69	39.96	9.27
64	Sample 2024-06-28 152020 GMT+0530 3	28.83	15.05	-13.78
70	Sample 2024-06-28 152928 GMT+0530 3	32.00	24.68	-7.32
71	Sample 2024-06-28 153019 GMT+0530 3	14.29	29.87	15.58

73	Sample 2024-06-28 103249 GMT+0530 3	14.36	26.72	12.36
74	Sample 2024-06-28 103439 GMT+0530 3	11.33	21.46	10.13
75	Sample 2024-06-28 103558 GMT+0530 3	12.14	22.73	10.59
81	Sample 2024-06-28 104212 GMT+0530 3	14.02	20.61	6.59
84	Sample 2024-06-28 104526 GMT+0530 3	29.77	14.12	-15.65
85	Sample 2024-06-28 104626 GMT+0530 3	30.56	18.79	-11.77
86	Sample 2024-06-28 104819 GMT+0530 3	38.56	16.09	-22.47
87	Sample 2024-06-28 104920 GMT+0530 3	39.62	23.17	-16.45
88	Sample 2024-06-28 105029 GMT+0530 3	45.23	23.17	-22.06
92	Sample 2024-06-28 110154 GMT+0530 3	40.19	26.17	-14.02
93	Sample 2024-06-28 110243 GMT+0530 3	36.80	27.07	-9.73
96	Sample 2024-06-28 110521 GMT+0530 3	35.10	17.45	-17.65
