# Supplementary material for: Use of near-infrared spectroscopy for screening the oil content, protein, phytic acid, glucosinolates, and fatty acid profile in oilseed Brassica species
Source: Front Nutr. 2025 Sep 2;12:1632421. doi: 10.3389/fnut.2025.1632421 (PMC12439716; doi:10.3389/fnut.2025.1632421)
Supplement: Supplementary file 11 [file Data_Sheet_11.pdf]

Stearic acid C:\RESULT Data\Workflows\Fatty acid table (R2) Stearic 10-2-25.qnt
Revision: 2 Last saved on: Mon Feb 10 12:38:42 2025
Printed on: Mon Feb 10 12:40:57 2025

Calculated vs Actual Plot - stearic

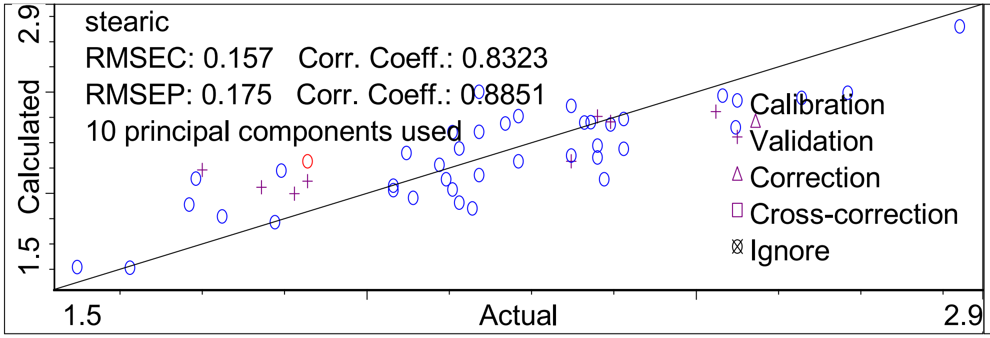


Difference vs Actual Plot - stearic

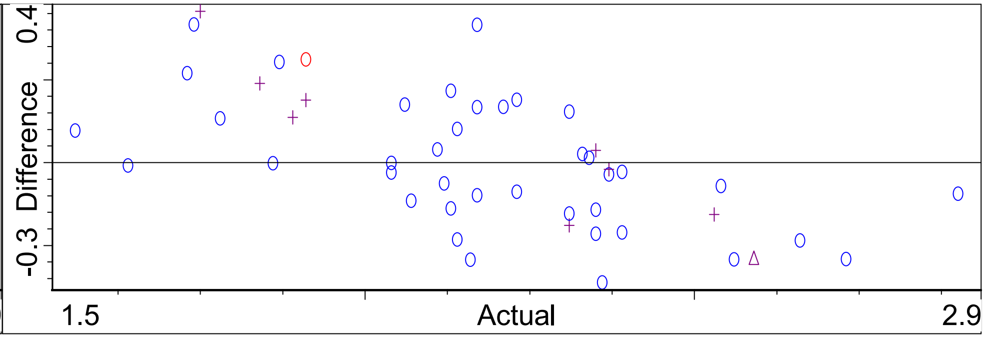


RMSEP: 0.175 Corr. Coeff.: 0.8851 10 principal components used
99.44% of variability described

**Calibration Results Table - stearic**

| Index | File Name | Spectrum Title | Usage | Actual | Calculated | Diff. x Path |
|---|---|---|---|---|---|---|
| 1 | aicrp 2018 1.spa | Sample 2024-06-28 105808 GMT+0530 | 0 | 1.91 | 2.16 | 0.25 |
| 2 | aicrp 2018 10 .spa | Sample 2024-06-28 151205 GMT+0530 | 1 | 1.75 | 2.12 | 0.37 |
| 5 | aicrp 2018 13.spa | Sample 2024-06-28 151557 GMT+0530 | 0 | 2.12 | 2.07 | -0.05 |
| 7 | aicrp 2018 15.spa | Sample 2024-06-28 151847 GMT+0530 | 0 | 1.78 | 1.89 | 0.11 |
| 11 | 2017 4.spa | Sample 2024-07-01 101909 GMT+0530 | 0 | 2.90 | 2.82 | -0.08 |
| 12 | 2017 5.spa | Sample 2024-07-01 102150 GMT+0530 | 0 | 2.14 | 1.95 | -0.19 |
| 15 | 2017 8.spa | Sample 2024-07-01 102410 GMT+0530 | 0 | 2.06 | 2.20 | 0.14 |
| 16 | 2017 9.spa | Sample 2024-07-01 102457 GMT+0530 | 0 | 2.16 | 1.93 | -0.23 |
| 17 | 2017 10.spa | Sample 2024-07-01 102555 GMT+0530 | 0 | 1.74 | 2.07 | 0.33 |
| 19 | 2017 14.spa | Sample 2024-07-01 102730 GMT+0530 | 0 | 2.17 | 2.50 | 0.33 |
| 21 | aicrp 2023 6 | Sample 2024-07-01 103633 GMT+0530 | 0 | 2.23 | 2.38 | 0.15 |
| 24 | aicrp 2023 11.spa | Sample 2024-07-01 104008 GMT+0530 | 0 | 2.39 | 2.37 | -0.02 |
| 27 | aicrp 2023 12.spa | Sample 2024-07-01 104434 GMT+0530 | 0 | 1.86 | 1.86 | -0.00 |
| 28 | aicrp 2023 3.spa | Sample 2024-07-01 104541 GMT+0530 | 0 | 2.33 | 2.35 | 0.02 |
| 31 | aicrp 2023 13.spa | Sample 2024-07-01 104821 GMT+0530 | 0 | 2.14 | 2.22 | 0.08 |
| 32 | aicrp 2023 5.spa | Sample 2024-07-01 104912 GMT+0530 | 0 | 2.56 | 2.33 | -0.23 |
| 33 | aicrp 2023 1.spa | Sample 2024-07-01 105049 GMT+0530 | 0 | 1.56 | 1.64 | 0.08 |
| 34 | aicrp 2023 18 | Sample 2024-07-01 105209 GMT+0530 | 1 | 2.35 | 2.38 | 0.03 |

Stearic acid C:\RESULT Data\Workflows\Fatty acid table (R2) Stearic 10-2-25.qnt
Revision: 2 Last saved on: Mon Feb 10 12:38:42 2025
Printed on: Mon Feb 10 12:40:57 2025

| | | | | | | |
|---|---|---|---|---|---|---|
| 35 | aicrp 2023 17.spa | Sample 2024-07-01 105458 GMT+0530 | 0 | 2.37 | 2.34 | -0.03 |
| 36 | aicrp 2023 19.spa | Sample 2024-07-01 105554 GMT+0530 | 0 | 2.66 | 2.47 | -0.19 |
| 38 | aicrp 2023 16.spa | Sample 2024-07-01 105755 GMT+0530 | 2 | 2.59 | 2.36 | -0.23 |
| 45 | aicrp 2023 22 r | Sample 2024-07-01 110952 GMT+0530 | 0 | 2.73 | 2.50 | -0.23 |
| 49 | aicrp 2023 14 r | Sample 2024-07-01 111339 GMT+0530 | 0 | 2.35 | 2.18 | -0.17 |
| 50 | aicrp 2023 24 r .s | Sample 2024-07-01 111438 GMT+0530 | 1 | 2.31 | 2.16 | -0.15 |
| 53 | aicrp 2023 26 r . | Sample 2024-07-01 111708 GMT+0530 | 0 | 2.34 | 2.35 | 0.01 |
| 57 | aicrp 2023 20 r . | Sample 2024-07-01 112144 GMT+0530 | 0 | 2.31 | 2.43 | 0.12 |
| 61 | aicrp 2023 7 r .s | Sample 2024-07-01 112847 GMT+0530 | 0 | 2.35 | 2.24 | -0.11 |
| 63 | aicrp 2023 4 r .s | Sample 2024-07-01 113054 GMT+0530 | 0 | 2.54 | 2.48 | -0.06 |
| 67 | aicrp 2018 18.spa | Sample 2024-06-28 152259 GMT+0530 | 0 | 1.73 | 1.95 | 0.22 |
| 69 | aicrp 2018 20.spa | Sample 2024-06-28 152639 GMT+0530 | 1 | 2.37 | 2.35 | -0.02 |
| 70 | aicrp 2018 21.spa | Sample 2024-06-28 152928 GMT+0530 | 0 | 1.64 | 1.63 | -0.01 |
| 71 | aicrp 2018 22.spa | Sample 2024-06-28 153019 GMT+0530 | 0 | 2.04 | 2.02 | -0.02 |
| 72 | aicrp 2021 1 samp | Sample 2024-06-28 102909 GMT+0530 | 0 | 2.36 | 2.07 | -0.29 |
| 74 | aicrp 2021 3.spa | Sample 2024-06-28 103439 GMT+0530 | 0 | 2.21 | 2.34 | 0.13 |
| 75 | aicrp 2021 4.spa | Sample 2024-06-28 103558 GMT+0530 | 0 | 2.17 | 2.30 | 0.13 |
| 76 | aicrp 2021 5.spa | Sample 2024-06-28 103658 GMT+0530 | 0 | 1.87 | 2.11 | 0.24 |
| 77 | aicrp 2021 6.spa | Sample 2024-06-28 103810 GMT+0530 | 1 | 1.89 | 2.00 | 0.11 |
| 79 | aicrp 2021 8.spa | Sample 2024-06-28 104016 GMT+0530 | 0 | 2.13 | 2.30 | 0.17 |
| 81 | aicrp 2021 10.spa | Sample 2024-06-28 104212 GMT+0530 | 0 | 2.17 | 2.09 | -0.08 |
| 82 | aicrp 2021 11.spa | Sample 2024-06-28 104332 GMT+0530 | 0 | 2.11 | 2.14 | 0.03 |
| 85 | aicrp 2021 14.spa | Sample 2024-06-28 104626 GMT+0530 | 0 | 2.04 | 2.04 | -0.00 |
| 86 | aicrp 2021 15.spa | Sample 2024-06-28 104819 GMT+0530 | 1 | 2.53 | 2.40 | -0.13 |
| 87 | aicrp 2021 16.spa | Sample 2024-06-28 104920 GMT+0530 | 0 | 2.39 | 2.22 | -0.17 |
| 89 | aicrp 2018 1.spa | Sample 2024-06-28 105808 GMT+0530 | 0 | 2.23 | 2.16 | -0.07 |
| 90 | aicrp 2018 2.spa | Sample 2024-06-28 105951 GMT+0530 | 0 | 2.07 | 1.98 | -0.09 |
| 92 | aicrp 2018 4.spa | Sample 2024-06-28 110154 GMT+0530 | 1 | 1.84 | 2.03 | 0.19 |
| 94 | aicrp 2018 6.spa | Sample 2024-06-28 110352 GMT+0530 | 0 | 2.13 | 2.02 | -0.11 |
| 95 | aicrp 2018 7.spa | Sample 2024-06-28 110435 GMT+0530 | 0 | 2.31 | 2.19 | -0.12 |
| 96 | aicrp 2018 8.spa | Sample 2024-06-28 110521 GMT+0530 | 1 | 1.91 | 2.06 | 0.15 |
| 3 | aicrp 2018 11.spa | Sample 2024-06-28 151404 GMT+0530 | 3 | 1.43 | 2.27 | 0.84 |
| 4 | aicrp 2018 12.spa | Sample 2024-06-28 151502 GMT+0530 | 3 | 2.40 | 1.99 | -0.41 |

Stearic acid   C:\RESULT Data\Workflows\Fatty acid table (R2) Stearic 10-2-25.qnt
Revision:  2    Last saved on:  Mon Feb 10 12:38:42 2025
Printed on:  Mon Feb 10 12:40:57 2025

| | | | | | | |
|---|---|---|---|---|---|---|
| 6 | aicrp 2018 14.spa | Sample 2024-06-28 151802 GMT+0530 | 3 | 1.62 | 2.17 | 0.55 |
| 8 | 2017 1.spa | Sample 2024-07-01 101644 GMT+0530 | 3 | 0.81 | 2.46 | 1.65 |
| 9 | 2017 2.spa | Sample 2024-07-01 101731 GMT+0530 | 3 | 0.99 | 2.50 | 1.51 |
| 10 | 2017 3.spa | Sample 2024-07-01 101818 GMT+0530 | 3 | 3.34 | 2.52 | -0.82 |
| 13 | 2017 6.spa | Sample 2024-07-01 102238 GMT+0530 | 3 | 1.00 | 2.34 | 1.34 |
| 14 | 2017 7.spa | Sample 2024-07-01 102324 GMT+0530 | 3 | 2.50 | 2.11 | -0.39 |
| 18 | 2017 13.spa | Sample 2024-07-01 102640 GMT+0530 | 3 | 1.20 | 2.09 | 0.89 |
| 20 | 2017 15.spa | Sample 2024-07-01 102812 GMT+0530 | 3 | 1.10 | 2.33 | 1.23 |
| 22 | aicrp 2023 8.spa | Sample 2024-07-01 103829 GMT+0530 | 3 | 3.23 | 2.47 | -0.76 |
| 23 | aicrp 2023 9 | Sample 2024-07-01 103923 GMT+0530 | 3 | 2.00 | 2.45 | 0.45 |
| 25 | aicrp 2023 10 | Sample 2024-07-01 104201 GMT+0530 | 3 | 0.63 | 2.41 | 1.78 |
| 26 | aicrp 2023 4.spa | Sample 2024-07-01 104338 GMT+0530 | 3 | 1.67 | 1.97 | 0.30 |
| 29 | aicrp 2023 14.spa | Sample 2024-07-01 104627 GMT+0530 | 3 | 3.02 | 2.46 | -0.56 |
| 30 | aicrp 2023 2 | Sample 2024-07-01 104720 GMT+0530 | 3 | 2.67 | 2.13 | -0.54 |
| 37 | aicrp 2023 7.spa | Sample 2024-07-01 105658 GMT+0530 | 3 | 2.77 | 2.34 | -0.43 |
| 39 | aicrp 2023 20.spa | Sample 2024-07-01 105857 GMT+0530 | 3 | 2.52 | 1.64 | -0.88 |
| 40 | aicrp 2023 9 r .s | Sample 2024-07-01 110402 GMT+0530 | 3 | 1.11 | 2.09 | 0.98 |
| 41 | aicrp 2023 17 r .s | Sample 2024-07-01 110546 GMT+0530 | 3 | 2.89 | 1.86 | -1.03 |
| 42 | aicrp 2023 17 r s | Sample 2024-07-01 110639 GMT+0530 | 3 | 1.20 | 2.10 | 0.90 |
| 43 | aicrp 2023 1 r .s | Sample 2024-07-01 110747 GMT+0530 | 3 | 1.15 | 1.87 | 0.72 |
| 44 | aicrp 2023 25 r .sp | Sample 2024-07-01 110839 GMT+0530 | 3 | 2.77 | 2.48 | -0.29 |
| 46 | aicrp 2023 19 r . | Sample 2024-07-01 111050 GMT+0530 | 3 | 1.15 | 2.27 | 1.12 |
| 47 | aicrp 2023 2 r .s | Sample 2024-07-01 111201 GMT+0530 | 3 | 3.23 | 2.36 | -0.87 |
| 48 | aicrp 2023 6 r s | Sample 2024-07-01 111251 GMT+0530 | 3 | 1.67 | 2.23 | 0.56 |
| 51 | aicrp 2023 11 r.sp | Sample 2024-07-01 111523 GMT+0530 | 3 | 0.56 | 2.20 | 1.64 |
| 52 | aicrp 2023 18 r . | Sample 2024-07-01 111616 GMT+0530 | 3 | 1.25 | 2.10 | 0.85 |
| 54 | aicrp 2023 12 r . | Sample 2024-07-01 111809 GMT+0530 | 3 | 0.56 | 2.36 | 1.80 |
| 55 | aicrp 2023 23 r . | Sample 2024-07-01 111901 GMT+0530 | 3 | 1.49 | 2.41 | 0.92 |
| 56 | aicrp 2023 21 r .s | Sample 2024-07-01 111955 GMT+0530 | 3 | 1.54 | 2.52 | 0.98 |
| 58 | aicrp 2023 16 r . | Sample 2024-07-01 112521 GMT+0530 | 3 | 3.01 | 2.21 | -0.80 |
| 59 | aicrp 2023 10 r . | Sample 2024-07-01 112629 GMT+0530 | 3 | 2.65 | 2.10 | -0.55 |
| 60 | aicrp 2023 8 r .s | Sample 2024-07-01 112725 GMT+0530 | 3 | 0.31 | 2.12 | 1.81 |
| 62 | aicrp 2023 5 r .s | Sample 2024-07-01 113001 GMT+0530 | 3 | 3.21 | 2.64 | -0.57 |

| | | | | |
|---|---|---|---|---|
| 64 | aicrp 2023 3 r .sSample 2024-07-01 113146 GMT+0530 3 | 3.14 | 2.51 | -0.63 |
| 65 | aicrp 2018 16.spa Sample 2024-06-28 152020 GMT+0530 3 | 1.49 | 2.30 | 0.81 |
| 66 | aicrp 2018 17.spa Sample 2024-06-28 152204 GMT+0530 3 | 1.35 | 1.90 | 0.55 |
| 68 | aicrp 2018 19.spa Sample 2024-06-28 152439 GMT+0530 3 | 0.92 | 2.38 | 1.46 |
| 73 | aicrp 2021 2.spa Sample 2024-06-28 103249 GMT+0530 3 | 2.66 | 2.15 | -0.51 |
| 78 | aicrp 2021 7.spa Sample 2024-06-28 103922 GMT+0530 3 | 1.22 | 2.17 | 0.95 |
| 80 | aicrp 2021 9.spa Sample 2024-06-28 104111 GMT+0530 3 | 2.87 | 2.55 | -0.32 |
| 83 | aicrp 2021 12.spa Sample 2024-06-28 104424 GMT+0530 3 | 2.54 | 2.20 | -0.34 |
| 84 | aicrp 2021 13.spa Sample 2024-06-28 104526 GMT+0530 3 | 2.64 | 2.21 | -0.43 |
| 88 | aicrp 2021 17.spa Sample 2024-06-28 105029 GMT+0530 3 | 2.81 | 2.17 | -0.64 |
| 91 | aicrp 2018 3.spa Sample 2024-06-28 110045 GMT+0530 3 | 1.57 | 2.05 | 0.48 |
| 93 | aicrp 2018 5.spa Sample 2024-06-28 110243 GMT+0530 3 | 1.92 | 2.44 | 0.52 |
| 97 | aicrp 2018 9.spa Sample 2024-06-28 110943 GMT+0530 3 | 0.80 | 2.49 | 1.69 |
